# Epigenetic regulation in the tumor microenvironment: molecular mechanisms and therapeutic targets

**DOI:** 10.1038/s41392-023-01480-x

**Published:** 2023-05-22

**Authors:** Jing Yang, Jin Xu, Wei Wang, Bo Zhang, Xianjun Yu, Si Shi

**Affiliations:** 1 Department of Pancreatic Surgery, Fudan University Shanghai Cancer Center, Shanghai, China; 2 Department of Oncology, Shanghai Medical College, Fudan University, Shanghai, China; 3 Shanghai Pancreatic Cancer Institute, Shanghai, China; 4 Pancreatic Cancer Institute, Fudan University, Shanghai, China

**Keywords:** Cancer microenvironment, Epigenetics

## Abstract

Over decades, researchers have focused on the epigenetic control of DNA-templated processes. Histone modification, DNA methylation, chromatin remodeling, RNA modification, and noncoding RNAs modulate many biological processes that are crucial to the development of cancers. Dysregulation of the epigenome drives aberrant transcriptional programs. A growing body of evidence suggests that the mechanisms of epigenetic modification are dysregulated in human cancers and might be excellent targets for tumor treatment. Epigenetics has also been shown to influence tumor immunogenicity and immune cells involved in antitumor responses. Thus, the development and application of epigenetic therapy and cancer immunotherapy and their combinations may have important implications for cancer treatment. Here, we present an up-to-date and thorough description of how epigenetic modifications in tumor cells influence immune cell responses in the tumor microenvironment (TME) and how epigenetics influence immune cells internally to modify the TME. Additionally, we highlight the therapeutic potential of targeting epigenetic regulators for cancer immunotherapy. Harnessing the complex interplay between epigenetics and cancer immunology to develop therapeutics that combine thereof is challenging but could yield significant benefits. The purpose of this review is to assist researchers in understanding how epigenetics impact immune responses in the TME, so that better cancer immunotherapies can be developed.

## Background

Chromatin is the DNA and histone protein macromolecular complex that supplies the scaffold for the packaging of our whole genome. It contains the genetic material of eukaryotic cells. The fundamental functional unit of chromatin is the nucleosome. It is composed of 147 DNA base pairs wrapped around an octamer of histones H2A, H2B, H3, and H4. All of the nucleosome components are susceptible to covalent alteration, which significantly modifies the structure and function of these key chromatin constituents, as revealed by research into the coordinated control of the nucleosome.

The term epigenetics was coined by Conrad Waddington to describe the process by which modifications to a cell’s phenotype can be passed down across generations without requiring a change to the DNA sequence. A consensus definition of epigenetics is lacking, and the definitions remain vague after decades of debate and research.^1^ Therefore, the word epigenetics will be used throughout this review to refer to chromatin-based activities that govern DNA-programmed processes.

Chromatin-modifying enzymes actively add to and remove modifications from DNA and histones in a highly controlled way. Currently, at least four different modifications to DNA and histones have been identified.^2,3^ These alterations can alter the structure of chromatin by modifying the noncovalent interactions between and within nucleosomes. In addition, they serve as docking sites for proteins with specific domains that may identify these alterations. These chromatin readers recruit other chromatin modifiers and remodeling enzymes to implement the changes.

Numerous oncogenes and tumor suppressor genes can accumulate mutations and epigenetic modifications that lead to cancer.^4,5^ Increasing evidence suggests that epigenetic alteration is involved in a number of tumor cell biological activities, such as proliferation, invasion, metastasis, and metabolic reprogramming.^6,7^ Malignant cell differentiation, proliferation, invasion, metastasis, and even medication therapy resistance is influenced by the interplay between malignant cells and the immediate environment influences, affecting how a tumor progresses.^6,8^ Recent research has demonstrated that epigenetics regulates immune cell activation and infiltration into TME, which may alter immunotherapy efficacy.^9,10^ Therefore, epigenetic alterations are potential tumor immunotherapy targets that can be employed in conjunction with treatments such as immune checkpoint inhibitors (ICIs) to significantly improve tumor patient survival and quality of life. Here, we present a current and comprehensive summary of epigenetic modification and associated immunological responses in the TME. In addition, the possibility of targeting epigenetic regulators in cancer immunotherapy is highlighted.^11^

### Epigenetic modifications

#### DNA methylation

The attachment of a methyl group to the 5-carbon of cytosine (5mC) in CpG dinucleotides was the first identified type of epigenetic alteration and is the most well-studied modification of chromatin.^12,13^ Telomeres, dormant X chromosomes, centromeres, and repetitive DNA sequences are common sites of DNA methylation.^14^ DNA methylation is involved in a wide variety of biological processes, such as X-chromosome inactivation, imprinting, and the maintenance of genomic stability.^15–18^ In cancer, global DNA hypomethylation was first observed experimentally ~30 years ago.^19^ Global DNA hypomethylation and hypermethylation of tumor suppressor gene promoters are hallmarks of cancer cells and key driver of carcinogenesis.^20^

DNA methylation is a dynamic process that can be influenced by writers, erasers, and readers. The DNA methyltransferase (DNMT) enzymes DNMT1, DNMT3A, and DNMT3B transfer a methyl group from S-adenosyl-l-methionine to the cytosine residue. DNMT1 is a maintenance methyltransferase that detects hemimethylated DNA created during DNA replication and methylates newly synthesized CpG dinucleotides whose parental strand partners are already methylated.^21^ DNMT3A and DNMT3B, despite their ability to methylate hemimethylated DNA, largely function as de novo methyltransferases to initiate DNA methylation during embryogenesis.^22^

**Fig. 1:**
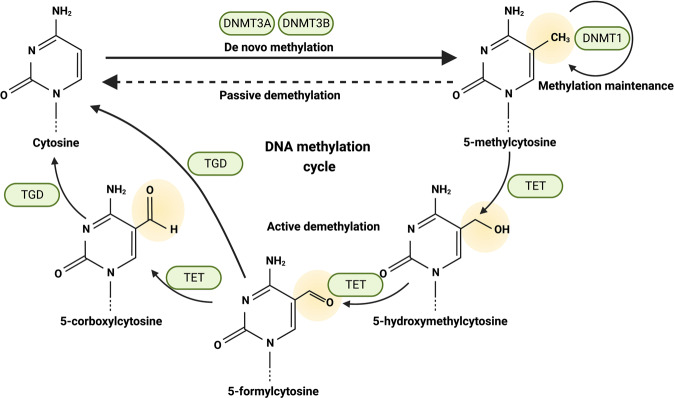
DNA methylation. DNA methylation is a dynamic process modulated by writers, erasers and readers. DNA methyltransferases (DNMTs) enzymes (“writers”) transfer a methyl group from S-adenosyl-L-methionine to the cytosine residue (5mC), including DNMT1, DNMT3A and DNMT3B. 5mC can be demethylated to 5-hydroxymethylcytosine (5hmC) via erasers. Indeed, 5hmC is iteratively oxidized to produce further oxidative derivatives, including 5-formylcytosine (5fC) and 5-carboxycytosine (5caC). The iterative oxidation reactions are performed by the ten-eleven translocation (TET1–3) family of proteins

5mC can be demethylated to 5-hydroxymethylcytosine (5hmC) via erasers. Indeed, 5hmC is iteratively oxidized to produce additional oxidative derivatives, including 5-formylcytosine (5fC) and 5-carboxycytosine (5caC). Iterative oxidation reactions are performed by the ten-eleven translocation (TET) family of proteins (Fig. 1). In mammalian DNA, the TET1–3 protein family is responsible for the catalytic conversion of 5mC into 5hmC. In addition, various oxidation derivatives, including 5fC and 5caC, are produced through the repeated oxidation of 5hmC by the TET family members.^6^ 5hmC is involved in transcriptional activation and inhibition, and TET proteins have been identified as having common activities.^3^

To coordinate these downstream regulatory processes, DNA methylation provides a platform for various methyl-binding proteins (“readers”), which regulate the crosstalk between DNA methylation, histone modifications, and chromatin architecture. MeCP2, the prototypical member of the methyl-CpG-binding domain (MBD) family of proteins (MBD1, MBD2, and MBD3), recruits histone-modifying enzymes, chromatin remodelers, and DNMTs to methylated CpGs involved in gene repression.^23–27^

#### Histone modification

Histone modifications are a class of post-translational modifications (PTMs) that impact chromatin structure and have been found to have a crucial influence on transcription and all DNA-template processes. Typically, marks are located on the N-terminal ‘tails’ of histones and contribute to nucleosome stability.^28^ At least 16 distinct histone PTMs, including acetylation, methylation, and phosphorylation, have been discovered to date. Histone modifications are dynamically regulated by proteins called “writers”, “readers”, and “erasers”. The transcriptional activation or repression of genes is affected by abnormal histone modifications, which also influence numerous processes, including DNA replication and recombination, and hence impair cell homeostasis and control tumor formation.^6,29^

##### Histone acetylation

Enzymes called histone acetyltransferases (HATs) add acetyl groups to the ε-amino group of lysine side chains. Acetyl-CoA is a cofactor for a number of enzymes with roles in transcription, chromatin structure, and DNA repair.^30^ The neutralization of lysine’s positive charge by acetylation may impair the electrostatic connection between positively charged histones and negatively charged DNA. Consequently, histone acetylation is frequently linked to a more “open” chromatin conformation.

Type-B HATs are primarily cytoplasmic; they acetylate free histones but not those already deposited into chromatin, and type-A HATs are primarily nuclear; they are divided into three subfamilies based on amino acid sequence homology and conformational structure: the Gcn5-related N-acetyltransferase family (GNATs), the MYST family (MOZ, Ybf2, Sas2, and TIP60), and the orphan family (CBP/EP300 and nuclear receptors).^31,32^

Histone deacetylases (HDACs) counteract the effects of HATs and restore the positive charge of the lysine side chains. HDACs are substrate-specific, meaning they can target not only histones but also nonhistone proteins such as HATs. HDACs are classified into four primary groups: class I (HDAC1, HDAC2, HDAC3, HDAC8), class IIa (HDAC4, HDAC5, HDAC7, HDAC9), class IIb (HDAC6, HDAC10), class III (sirtuin1-7), and class IV (HDAC11) HDACs. Only class III HDACs require nicotinamide adenine dinucleotide(NAD), while classes I, II, and IV HDACs require Zn^2+^.^33^

In addition, acetylation can serve as a signal in chromatin that is recognized by “readers” (a subset of bromodomain proteins called bromodomain and extraterminal domain (BET)). The BET family comprises of four members with a common architecture and structural design: BRD2, BRD3, BRDT, and BRDT. Targeting the BET bromodomains with epigenetic-based drugs is thus likely to be a potential cancer strategy. In addition, BET proteins are involved in a number of essential processes, including transcription initiation, transcription elongation, cell-cycle progression, DNA damage regulation, and telomere regulation.^34–37^

##### Histone methylation

On the side chains of lysine, arginine, and histidine residues, histones can be methylated without changing the total charge of the molecule. Monomethylated, dimethylated, and di-asymmetrically methylated forms of arginine can exist, as can monomethylated, dimethylated, and trimethylated forms of lysine. Among these types of methylation, histone lysine methylation has received the most attention. SUV39H1 was the first histone lysine methyltransferase (HKMT) targeting histone 3 lysine 9 (H3K9) to be found.^38^ HKMTs catalyze the transfer of a methyl group from S-adenosine methionine (SAM) to the ε-amino group of lysine. Remarkably, except for the Dot1 enzyme methylating H3K79, all HKMTs that methylate N-terminal lysine possess an enzymatically active SET domain. Histone lysine methyltransferases (HKMTs) are relatively specific. Histone 3 lysine 9 (H3K9) can be trimethylated (H3K9me3) from a monomethylated (H3K9me1) state by KMT1A/B, or it can be methylated to a dimethylated (H3K9me2) form by the H3K9 methyltransferase KMT1C (also known as G9a), with a preference for monomethylation to dimethylation.^39,40^ Different methylation sites have different effects. Examples of sites of histone modifications that are associated with euchromatin activity include H3K4, H3K36, and H3K79, while sites of histone modifications such as H3K9, H4K20, and H3K27 are associated with heterochromatin.^41^ Diverse methylation statuses on the same residue also have different functional implications. For example, trimethylation of H3K9 is associated with transcriptional repression, whereas monomethylation of H3K9 is present in actively transcribed genes.

In 2004, lysine-specific demethylase 1 (LSD1) was discovered to use FAD as a cofactor to reverse lysine methylation.^42^ JMJD2 was the first identified tri-methyl lysine demethylase with a distinct catalytic mechanism distinct from that of LSD1, employing Fe (II), alpha-ketoglutaric acid, and a free radical attack mechanism.^43^ JMJD2 demethylates H3K9me3 and H3K36me3. Demethylases, similar to histone methyltransferases, have a high substrate selectivity. They are also sensitive to the degree of lysine methylation; for example, certain enzymes can only demethylate monomethylated and dimethylated substrates, whereas others can demethylate all three forms of methylated lysine.

##### Histone phosphorylation

Similar to histone acetylation, the phosphorylation of histones is a highly dynamic process that is reciprocally regulated by protein kinases and protein phosphatases. It primarily, but not entirely affects serines, threonines, and tyrosines in the N-terminal tails of histones. Protein kinases and phosphatases, which add and remove the modification, respectively, work together to control the overall level of the modification.^44^ In general, histone phosphorylation sites are related to transcriptional regulation and in chromatin condensation.^45^ Most phosphorylation sites on histones are located in the N-terminal tails. However, there are sites within the main regions. One such instance is the phosphorylation of H3Y41 mediated by the nonreceptor JAK2.^46^

Except for the above three histone modifications, there are a variety of less prevalent and atypical PTMs, such as histone ubiquitination, ADP-ribosylation, deamination, and O-GlcNAcylation, etc., which have been reviewed in detail in refs. ^47,48^

#### RNA modification

##### N^6^-methyladenosine

The m^6^A modification appears mostly on the common sequence 5’-RRACH-3’ (R = A or G and H = A, C, or U),^49–51^ and the modification is mainly localized near a stop codon in a 3′ untranslated regions (3’UTRs) within a lengthy internal exons.^52–54^ Dynamic and reversible, the m^6^A modification process is mediated by m^6^A methyltransferases (writers), m^6^A demethylases (erasers), and m^6^A-binding proteins (readers).^55^

The dynamic process involved in the m^6^A deposition is regulated by a methyltransferase complex (writer). As the first known m^6^A methyltransferase and a major catalytic subunit, methyltransferase-like 3 (METTL3) binds S-adenosylmethionine (SAM) and transfers methyl groups from SAM to adenine bases in RNA.^49,56,57^ Methyltransferase-like 14 (METTL14) is necessary for the identification of RNA substrates and forms a stable heterodimer with METTL3, hence increasing the complex’s catalytic activity.^51,58,59^ As the main regulatory component of the complex, Wilms’ tumor 1-associating protein (WTAP) participates in the localization of METTL3-METTL14 heterodimers to nuclear speckles, thereby facilitating m^6^A modification.^60^ Furthermore, the complex includes zinc-finger CCCH domain-containing protein 13 (ZC3H13), RNA-binding motif protein 15 (RBM15), KIAA1429 (also called VIRMA), and its paralog RBM15B. It has been reported that KIAA1429 recruits and guides the localization of m^6^A methylation to the 3’ UTRs and close to a stop codon.^58,61^ ZC3H13 is a novel cofactor that binds with other components (such as RBM15 and WTAP) to regulate nuclear m^6^A modification.^62^ RBM15/15B is also crucial for the recruitment of writers to target sites.^63,64^ Recently, studies have shown that ZCCHC4,^65^ METTL5^66,67^ and METTL16^68^ can function as methyltransferases and thus contribute to the m^6^A modification of some small nuclear RNAs (snRNAs), noncoding RNAs (ncRNAs) and pre-mRNAs.

Demethylases (erasers) remove methyl groups from N6 adenosine. Two primary erasers are fat mass and obesity-associated protein (FTO)^69^ and alpha-ketoglutarate-dependent dioxygenase alkB homolog 5 (ALKBH5).^70^ These two proteins both eliminate the m^6^A mark from RNA to reverse the m^6^A modification. Moreover, alkB homolog 3 (ALKBH3) has been shown to enhance protein synthesis in cancer cells by mediating tRNA demethylation.

**Fig. 2:**
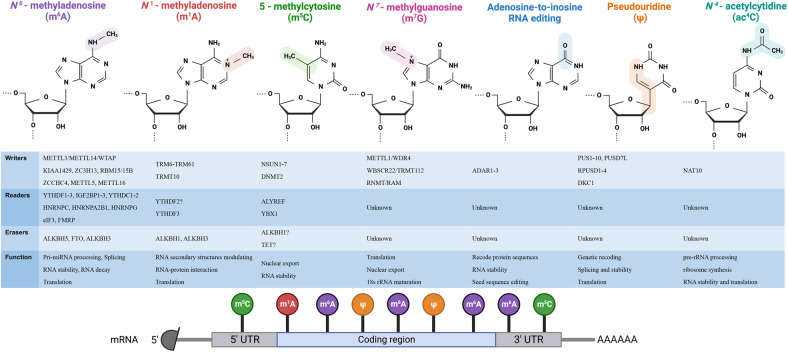
Internal RNA modifications. The main players in the deposition, removal and downstream recognition of the modification are listed together with the effect of modifications on base pairing. ADAR adenosine deaminase acting on double-stranded RNA, ADAT adenosine deaminase acting on transfer RNA, ALKBH alkB homolog, CTU cytoplasmic transfer RNA 2-thiolation protein, DKC1 dyskerin pseudouridine synthase 1, DNMT2 DNA methyltransferase-like2, ELP elongator complex protein, FTO fat mass and obesity-associated protein, m^1^A N1-methyladenosine, m^6^A N6-methyladenosine, m^5^C 5-methylcytosine, m^7^G 7-methylguanosine, METTL methyltransferase-like, NSUN NOL1/NOP2/SUN domain family member, PUS pseudouridine synthase, RNMT RNA guanine-7 methyltransferase, RPUSD RNA pseudouridine synthase domain-containing protein, TRM6 transfer RNA methyltransferase non-catalytic subunit 6, TRM61 transfer RNA methyltransferase catalytic subunit 61, TRMT10 transfer RNA methyltransferase 10, tRNA transfer RNA, WBSCR22 Williams–Beuren syndrome chromosomal region 22 protein, YTHDC YTH domain-containing, YTHDF YTH domain-containing family, ZCCHC4 zinc-finger CCHC domain-containing protein 4, NAT10 N-acetyltransferase 10

The m^6^A-binding proteins (readers) recognize and interact with the m^6^A marks on target transcripts.^71^ Different readers can drive multiple biological processes, such as mRNA splicing, export and stability, miRNA biogenesis, translation efficiency, and RNA structure switching. The YTH domains include the YTH domain family proteins 1, 2, and 3 (YTHDF1, YTHDF2^72,73^, and YTHDF3^74,75^) and YTH domain-containing proteins 1 and 2 (YTHDC1^76,77^ and YTHDC2^78^). In addition, insulin-like growth factors (IGF2BP1-3) are critical for enhancing mRNA stability.^79^ Furthermore, other readers, such as heterogeneous nuclear ribonuclease (HNRNP) family members (HNRNPA2B1,^80,81^ HNRNPC, HNRNPG,^82^ eukaryotic translation initiation factor 3 (eIF3), and fragile X mental retardation protein (FMRP), have also been demonstrated to perform a range of biological functions^83^ (Fig. 2).

##### N1-methyladenosine

In contrast to m^6^A marks, the N1-methyladenosine (m^1^A) marks show significantly lower abundance in mammalian tissues. Recent in-depth studies have shown that m^1^A modification is widely distributed throughout the whole transcriptome. Through electrostatic effects, the m^1^A mark can modulate RNA secondary structures and RNA‒protein interactions. m^1^A is enriched at translation start sites of mRNAs (5’ UTR) and in multiple regions of tRNAs, where it can upregulate translation and mediate a variety of corresponding biological processes.^84–86^

The tRNA methyltransferase catalytic subunit 6 (TRM6)–tRNA methyltransferase catalytic subunit 61 (TRM61) complex (TRM6–TRM61) is the only known methyltransferase capable of catalyzing the addition of N1-adenosine in mRNAs.^87^ Moreover, this complex and tRNA methyltransferase 10 homolog A (TRM10) can both direct the m^1^A modification of tRNAs.

AlkB homolog 3 (ALKBH3) is critical for removing the methyl group from m^1^A modification in mRNAs.^88^ In tRNAs, alkB homolog 1 (ALKBH1) ^89^ and ALKBH3^90^ can both modulate tumor progression by catalyzing m^1^A modification. Biochemical assays have indicated that YTHDF2 binds to m^1^A; therefore, YTHDF2 may be a potential m^1^A “reader”, a supposition that needs to be further verified.^91^ Recently, Zheng et al. identified YTHDF3 as the m^1^A “reader” by mass spectrometry^92^ (Fig. 2).

##### 5-Methylcytosine

5-Methylcytosine (m^5^C) carries a methyl group at the cytosine C5 position. The known writers to date include DNA methyltransferase 2 (DNMT2) and NOP2/Sun domain family members 1-7 (NSUN1-7).^93–95^ As the most researched methyltransferase, NSUN2 catalyzes the m^5^C modification of mRNAs, tRNAs, rRNAs, mitochondrial tRNAs (mt-tRNAs), long ncRNAs (lncRNAs), ncRNAs, and other RNAs.^96–100^ In addition, DNMT2 has been widely studied and is known to methylate mRNAs and tRNAs in anticodon loops.^101^ NSUN1 and NSUN5 localize to the nucleolus and methylate conserved residues in 28 S rRNA.^102–104^ NSUN3 is critical for 5-formylcytidine (f^5^m) biogenesis in mt-tRNAs.^105,106^ As a mitochondrial protein, NSUN4 is essential for small rRNA subunit methylation. NSUN6 specifically methylates Thr and Cys in tRNA at position C72.^107^ To determine the exact function of NSUN7, much research is needed.

To date, the identity of m^5^C erasers is controversial, and the research in this area is not mature. The ten-eleven translocator family (TET) has been suggested to oxidize m^5^C to form 5-hydroxymethylcytosine (hm^5^C) on mRNA.^108,109^ ALKBH1 can oxidize m^5^C to form 5-formylcytidine (f^5^C) at a wobble position in mt-tRNAs.^106^

Aly/REF export factor (ALYREF, an mRNA transport adapter, also called THOC4) has been reported to be an m^5^C reader that regulates mRNA export.^99^ Y-box binding protein 1 (YBX1) has been identified as a unique m^5^C-binding protein that modulates the cytoplasmic stability of mRNA^110^ (Fig. 2).

##### N7-methylguanosine

Currently, research on N7-methylguanosine (m^7^G) is in the early stages. The m^7^G modification has been detected on tRNAs, rRNAs, miRNAs, miRNA precursors and mRNAs.^111–115^ In addition, m^7^G modification is a dynamic process that is upregulated under stress conditions.

The identified m^7^G methyltransferases in mammals include methyltransferase-like 1/WD repeat domain 4 (the METTL1/WDR4 complex),^116^ Williams–Beuren syndrome chromosome region 22/tRNA methyltransferase activator subunit 11–2 (the WBSCR22/TRMT112 complex)^117,118^ and RNA guanine-7 methyltransferase/RNMT-activating miniprotein (the RNMT/RAM complex).^119^ Among these complexes, METTL1 binds with its cofactor WDR4 to deposit an m^7^G mark on tRNAs, miRNAs, and mRNAs; thus, the METTL1/WDR4 complex can impact tRNA function, promote miRNA biogenesis and regulate translation efficacy.^114,115,120,121^ The WBSCR22/TRMT112 complex is critical for rRNA m^7^G modification.^117,118^ The RNMT/RAM complex deposits an m^7^G mark at the 5’ caps of mRNAs, thus influencing RNA export and translation efficacy^122^ (Fig. 2).

##### Adenosine-to-inosine RNA editing

In recent years, A-to-I RNA editing has been shown to correlate with tumor formation and progression. The adenosine deaminase acting on RNA (ADAR) family is responsible for the A-to-I RNA editing process, which involves deaminating adenosine to create inosine on double-stranded RNAs (dsRNAs).^123,124^

A-to-I editing in coding areas can recode protein sequences, generate novel protein isoforms, and promote proteome diversity because inosine residues are misinterpreted as guanosine by cellular machinery. These recoding events have become a focus of in research in recent years. Notably, most of the A-to-I RNA editing occurs in noncoding transcriptome regions. Noncoding RNA editing plays a variety of functional roles. For instance, it can change the pre-mRNA splicing pattern, thereby introducing new protein isoforms. The ADAR family comprises three known members in mammals, ADAR1-3. Catalytic deaminase domains and dsRNA-binding domains (dsRBDs) are present in each of these proteins. Additionally, ADAR1 carries Z-DNA-binding domains (ZDBDs). It has been found that ADAR1 and ADAR2 are expressed almost everywhere, whereas ADAR3 is mostly found in the brain^125–127^ (Fig. 2).

##### Pseudouridine

Pseudouridine (Ψ) is the C5-glycoside isomer of uridine. In the regular pyrimidine nucleosides, the C-1’ atom of the pentose forms a glycosidic connection with the N1 atom of the heterocyclic ring. However, in the pseudouracil nucleoside, the C-1’ atom of the pentose is bonded to the C5 atom of the heterocyclic pentose.^128–130^ As the first identified posttranscriptional modification and one of the most abundant, Ψ has been found in most types of RNAs, including rRNAs, tRNAs, miRNAs, lncRNAs, mRNAs, and snRNAs.^131–135^

Ψ synthase (also called pseudouridine synthase, PUSs) is a writer that catalyzes the conversion of uridine to Ψ. In eukaryotes, PUSs include PUS1-4, PUS6-7, PUS7L, PUS9-10, and RPUSD1-4.^136,137^ Another writer is dyskerin (DKC1).^138^ There are two mechanisms involved in pseudouridylation: an RNA-dependent mechanism and an RNA-independent mechanism. The RNA-independent mechanism involves direct recognition and catalysis by PUSs. Another RNA-dependent form of pseudouridylation depends on box H/ACA small ribonucleoproteins (snoRNPs),^130^ which are composed of a box H/ACA snoRNA and four core proteins: DKC1, nucleolar protein 10 (Nop10), nonhistone protein 2 (Nhp2) and glycine–arginine-rich protein 1 (Gar1). The complex is critical for recognizing substrates, with DKC1 showing catalytic activity (Fig. 2).

##### N^4^-acetylcytosine

Another conserved modification in cytidine is N4-acetylcytosine (ac^4^C; acetylation of the N4 position of cytosine), which is the only acetylation event known to occur in eukaryotic RNA.^139,140^ In rRNA, ac^4^C is located in helices 34 and 45 close to the decoding site of mammalian 18 S rRNA; in eukaryotic tRNA, it is found in the D-stem of tRNASer/Leu.^141–144^ The deposition of ac^4^C sites in mRNA occurs predominantly in the CDS region and partly in the 5’UTR.^145^ Currently, N-acetyltransferase 10 (NAT10), an important ATP-dependent RNA acetyltransferase, is regarded as the only “writer” of ac^4^C.^146^ Two extra proteins are necessary for the addition of ac^4^C to human rRNA or tRNA. The first is U13, a box C/D snoRNA that aids in the proper folding of pre-rRNA and is therefore essential for 18 S rRNA acetylation.^141^ The RNA adapter protein THUMP domain-containing 1 (THUMPD1) has a distinctive RNA-binding motif and can cooperate with NAT10 in tRNA acetylation.^141,144^

Ac^4^C has been shown to control pre-rRNA processing and ribosome production for 18 S rRNA and affect translation; promote tRNA stability; increase mRNA stability; and promote protein translation in mRNA CDS.^141,147–150^ As it is a recently discovered RNA modification, the regulators and molecular activities of ac^4^C are mostly unknown; hence additional research is required (Fig. 2).

#### Chromatin remodeling

The chromatin-remodeling complex depends on the energy generated by ATP hydrolysis to perform its remodeling function, and the core subunit is an ATPase-catalyzing subunit. It is possible to classify mammalian chromatin-remodeling complexes into four broad classes according to their biological function and constituent proteins: the switching defective/sucrose nonfermenting (SWI/SNF) family, the imitation SWI (ISWI) family, the nucleosome remodeling and deacetylation (NuRD)/Mi-2/chromodomain helicase DNA-binding (CHD) family, and the inositol requiring 80 (INO80) family.^151,152^

To achieve an active chromatin state, SWI/SNF complexes (also known as BAF (BRG1-associated factors) complexes) accelerate the ejection and insertion of histone octamers and facilitate the sliding movement of nucleosomes.^153^ SWI/SNF complexes are made up of one of two mutually exclusive catalytic ATPase subunits (SMARCA2 (Brahma or BRM) or SMARCA4 (BRM/SWI2-related gene 1, or BRG1)); a set of widely expressed and conserved core subunits (SMARCB1 (SNF5, INI-1, or BAF47), SMARCC1 (BAF155) and SMARCC2 (BAF170)) and a significant number of lineage-restricted subunits, which are frequently encoded by multigene families.^154,155^

Most eukaryote ISWI family remodelers contain 1–2 catalytic subunits and specialized attendant proteins. By regulating the distances between nucleosomes, certain ISWI family complexes (ACF, CHRAC) aid in chromatin assembly and transcriptional repression. Nonetheless, some complexes (NURF) can randomize spacing, which can improve RNAPII activation. This demonstrates the variety that can be generated by subunits.^156^

Two tandemly organized chromodomains are found at the N-terminus of the catalytic subunit in remodelers of the CHD family.^157^ Frequently, accompanying proteins have DNA-binding domains as well as PHD, BRK, CR1-3, and SANT domains. The transcription rate can be increased by the action of specific CHD remodelers that either slide nucleosomes or remove them. Others, such as the vertebrate Mi-2/NuRD (nucleosome remodeling and deacetylase) complex, play repressive roles.^158^

Orthologs of Ino80, Rvb1-2, Arp4-5, Arp8, Ies2, and Ies6 can be found in purified human INO80 complexes, alongside four other subunits that are exclusive to this family of remodelers.^159^ Through many pathways, INO80 can increase transcriptional activation and DNA repair.^160^

#### Noncoding RNAs

Small ncRNAs (sncRNAs) and lncRNAs (>200 bp) are the two forms of ncRNAs and cannot be translated into proteins. SncRNAs include small nucleolar RNAs (snoRNAs), microRNAs (miRNAs), small interfering RNAs (siRNAs), PIWI-interacting RNAs (piRNAs), extracellular RNAs (exRNAs), and circular RNAs (circRNAs).^161–163^ Aberrant expression of ncRNAs has been identified to be associated with carcinogenesis and metastasis in various cancers through epigenetic regulation.^162,164,165^ NcRNAs exert critical roles in regulating gene expression via promoting the complex formation and protein interactions during transcriptional or translational repression.^166,167^

One of the most extensively investigated ncRNAs is a single-stranded, approximately 20-base-long miRNA that mediates the degradation and cleavage of messenger RNAs by targeting the 3′-untranslated region (3’UTR), thereby preventing translation.^163,168^ Thousands of miRNAs have been discovered to act as tumor suppressors or promoters over the past few years.^62,169,170^ In addition, lncRNAs have also been found to regulate several biological processes such as tumor cell proliferation, invasion, migration and TME remodeling by modulating mRNA progression and transcription.^171–175^ CircRNAs are the result of mRNA precursor back splicing.^176^ They are endogenous ncRNAs that lack 3′ and 5′ ends and are structurally extremely conserved and stable. Several circRNAs have also been implicated in tumor suppression and carcinogenesis.^177–180^ Significantly, competing endogenous RNAs (ceRNAs) are posttranscriptional regulatory factors that have been widely studied in recent years. CeRNAs (most commonly lncRNAs and circRNAs) can influence miRNA-induced gene silencing by binding microRNA response elements (MREs) with miRNAs, thereby modulating tumor progression.^181,182^

### Modulation of epigenetically modified tumor cells in the TME

#### DNA methylation

Through extensive studies, 5mC-driven events have been verified to be important molecular mechanisms of carcinogenesis.^183–185^ Liu and colleagues established three 5mC modification models according to the clinical properties of 21 5mC modulators to analyze the potential role of 5mC regulators in the TME. For the purpose of assessing tumor mutation burden, ICI responsiveness, and prognostic characteristics, the 5mC score was established. The research team found that both the therapeutic benefit and immune cell infiltration were increased in patients with a low 5mC score.^186^ Similar studies also found that the group with a high 5mC score was associated with limited cancer immunotherapy sensitivity, and a low 5mC score was associated with a better response to immunotherapy in patients with bladder cancer (BLCA) and lung squamous cell carcinoma (LUSC).^187^ These results suggest that the 5mC score may serve as a biomarker for predicting the prognosis of cancer patients and gauging the efficacy of cancer treatments.

Several studies have demonstrated that DNA methyltransferase inhibitors (DNMTis) can enhance immunological responses. For example, dsRNA from endogenous retroviruses can cause an interferon response, macrophage polarization into an M1-like phenotype and subsequent T-cell activation, the release of tumor-associated antigens (TAAs), and major histocompatibility complex (MHC) class-I antigen presentation to immune cells in ovarian cancer (OC) and other solid tumors.^188–192^ On the basis of the aforementioned observations, Sara Moufarrij et al. found that DNMTi combined with HDAC6i could enhance the antitumor immune signal of OC cells. Treatment with HDAC6i and DNMTi led to amplification of the type I interferon response, increased cytokine and chemokine expression and upregulated MHC class-I antigen presentation complex expression. Treatment of mice carrying ID8 Trp53^-/-^ OC with the HDAC6i and DNMTi resulted in an increase in IFNg^+^CD8, natural killer (NK) and NKT cells, and reversed the immunosuppressive TME by reducing myeloid-derived suppressor cells (MDSCs) and PD1^hi^ CD4^+^ T cells, ultimately resulting in a beneficial effect on the TME of OC.^193^

For 5hmC, TET1 expression has been shown to have a significant negative correlation with NF-κB, and TET1 inhibition is associated with significant immune cell infiltration. By binding to its consensus sequence in the TET1 promoter, p65 suppresses TET1 expression in breast cancer cells upon NF-κB activation; similar results have been reported in thyroid cancer, lung cancer, and melanoma.^194^ TET2 mutations are linked to myeloid malignancies, and research by Ko et al. demonstrated that these mutations reduce the enzyme’s catalytic activity. When compared to bone marrow samples from healthy controls, genomic DNA from patients with TET2 mutations consistently showed low amounts of 5hmC. TET2 deficiency in mouse hematopoietic progenitors also skewed their development toward monocyte/macrophage lineages in culture. Myeloid cancers may benefit from 5hmC measurement as a diagnostic and prognostic tool may be beneficial for the personalization of treatments and evaluation of anticancer therapy efficacy in patients with myeloid cancers.^195^

**Fig. 3:**
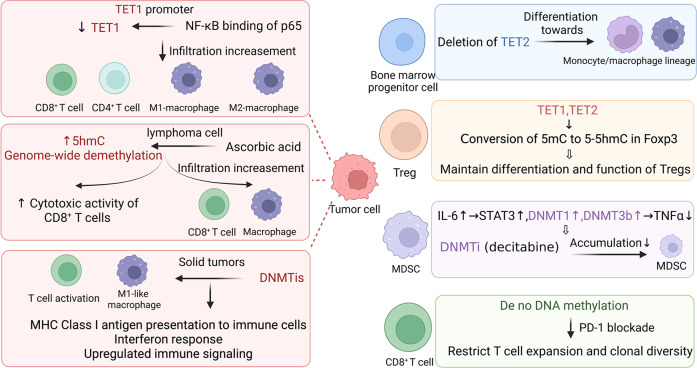
The main mechanisms by which DNA methylation remodels the TME. Aberrant DNA methylation of relevant genes in tumor cells has various effects in reprogramming the TME

Lymphoma cells treated with ascorbic acid (AA) have been shown to undergo genome-wide demethylation and have increased expression of endogenous retroviral elements. The results of both in vitro and in vivo studies have demonstrated that AA increases the level of 5hmC in CD8^+^ T cells and improves their cytotoxic activity. High-dose AA therapy in combination with anti-PD1 therapy dramatically reduced tumor growth in a mouse model of lymphoma, in comparison to the effects of either drug alone. In addition to increasing granzyme B synthesis by cytotoxic cells (cytotoxic T cells and NK cells) and IL-12 production by antigen-presenting cells, combination therapy also dramatically increased intratumoral infiltration of CD8^+^ T lymphocytes and macrophages^196^ (Fig. 3).

#### Histone modification

##### Histone acetylation

Considering the crucial role of histone acetylation (HA) crucial role in regulating chromatin shape, DNA repair, and gene expression,^197,198^ Xu et al. investigated the possible functions of HA regulators in TME cell invasion, drug sensitivity, and immunotherapy. Three HA patterns (low, medium, and high HAscore) were identified. High-risk hepatocellular carcinoma (HCC) was more likely to show enrichment of cancer-related malignant pathways and to have extensive infiltration of immunosuppressive cells such as regulatory T cells (Tregs) and MDSCs than HCC in the low-risk group based on the HAscore. The HAscore was closely associated with antitumor drug sensitivity, and the response rate to programmed death ligand 1 (PD-L1) and PD1 blockade was significantly greater in the group with the lowest HAscore.^199^ Examining the relationships between HA regulators and the potential clinical utility of HA regulators in HCC treatment and improving patient outcomes are primary goals of future studies. In B-lymphoma cells, mutation or knockdown of CREBBP or EP300 decreases H3K27 acetylation, downregulates FBXW7 expression, and activates the NOTCH pathway and downstream CCL2/CSF1 expression, leading to tumor cell proliferation and tumor-associated macrophage (TAM) polarization toward the M2 phenotype. Consistent results have also been obtained in B-lymphoma murine models.^200^

**Fig. 4:**
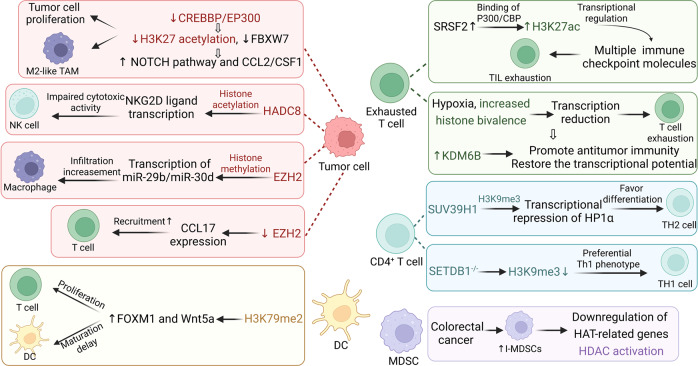
The main mechanisms by which histone modification remodels the TME. Aberrant histone modification of relevant genes in tumor cells has various effects in reprogramming the TME

In a recent study, the histone deacetylase HDAC8 was implicated in the modulation of the glioma immune response. The authors found that inhibiting HDAC8 with a specific inhibitor, PCI-34051, reduced tumor volume in glioma mouse models. HDAC8 regulates human and mouse glioma cell viability and tumor migration through a-tubulin acetylation. HDAC8 supports the hypoimmunogenic TME to regulate microglia phenotypes and regulate gene transcription of NKG2D ligands, thereby inhibiting NK cells mediated cytotoxic activity. Collectively, these results prove that HDAC8 is critical for glioma growth and the TME, and enable a deeper understanding of the molecular basis of glioma immune evasion^201^ (Fig. 4).

##### Histone methylation

By catalyzing H3K27me3 modification, enhancer of Zeste homolog 2 (EZH2) induces chromatin condensation, hence silencing specific genes epigenetically.^202^ Using differential expression analysis and a predictive model, Du et al. discovered that EZH2 expression in prostate cancer (PCa) is associated with DNA methylation alterations, TME, and immune-related genes. PCa patients with low EZH2 expression may be more sensitive to immunotherapy. The study revealed that EZH2 may be an effective predictor of PCa prognosis and immune response.^203^ Similar results of histone lysine methylation (HLM) regulators ((EZH2, NSD2, and KMT5C) found in an analysis of The Cancer Genome Atlas (TCGA)-PRAD dataset.^204^ Another study also observed a strong connection between EZH2 expression and macrophage infiltration in breast cancer, suggesting that modulating epigenetic regulation to control macrophage activation is a potential therapeutic strategy for breast cancer.^205^

The role of total histone H4 methylation (H4M) modification in the TME and immune regulation in HCC has also been recently reported. Analysis showed that the H4M modification model could predict the TME infiltration, tumor heterogeneity, prognosis and so on. The group with low H4Mscore was associated with better response to anti-PD1/L1 and anti-CTLA4 immunotherapy, as well as better survival outcomes. Hence, analyzing the H4M modification patterns in individual tumors may aid in the development of more effective immunotherapy strategies.^206^ Tazemetostat, an inhibitor of EZH2, has been developed for the treatment of B-cell lymphomas. A recent study suggests that tazemetostat may activate the anti-lymphoma response and promote T-cell recruitment by upregulating CCL17 expression in B-cell lymphoma cells, which provides a basis for its use in combination with immunotherapy.^207^ CD8^+^ effector T (Teff) cell development and polyfunctionality are disrupted and the propensity for terminal differentiation is increased after conditional knockout or shRNA-mediated deletion of Ezh2. However, methyltransferase inhibitors have not been used in a controlled setting to confirm these characteristics^208,209^ (Fig. 4).

#### RNA modifications

##### N6-methyladenosine

Growing evidence indicates that the m^6^A alteration is important for many biological functions, such as the DNA damage response,^210^ pluripotency,^211^ embryonic development,^212^ cell reprogramming,^213^ and circadian rhythm regulation.^214^ Moreover, an increasing number of studies have identified that the m^6^A mark is related to several malignant tumor processes, including tumorigenesis,^215^ proliferation,^216,217^ invasion,^218^ and metastasis.^219,220^ Through in-depth research, a growing number of investigations have demonstrated that m^6^A modulators are closely related to tumor immune responses and immune checkpoint blockade (ICB) efficacy. Important information on how the m^6^A alteration influences the immune cell response in the TME is highlighted here.

*Role of m*^*6*^*A writers*: T cells, which mature and migrate to peripheral organs, are the backbone of the adaptive immune system.^221,222^ Both CD4^+^ T cells and CD8^+^ T cells, distinguished by their respective cell-surface receptors, play critical roles in tumor cell destruction.^223,224^ Ni et al. revealed elevated METTL3 expression in bladder cancer, which was found to be essential for regulating RNA stability and the immune checkpoint PD-L1 expression, thereby inducing resistance to CD8^+^ T-cell cytotoxicity.^225^ Wan et al. obtained similar findings: METTL3 increased the PD-L1 expression in an m^6^A-IGF2BP3-dependent manner.^226^ Depletion of METTL3/14 increased the number of cytotoxic tumor-infiltrating CD8^+^ T cells, increased IFN-γ, CXCL9, and CXCL10 secretion in the TME and enhanced the anti-PD1 treatment response in mismatch-repair-proficient or microsatellite instability-low (pMMR-MSI-L) colorectal cancer (CRC) and melanoma by stabilizing the Stat1 and Irf1 mRNA in a manner mediated by YTHDF2. The novel regulatory mechanism of METTL3- and METTL14-mediated epigenetic modification indicates potential targets in cancer immunotherapy.^227^ Recently, new evidence regarding the regulation of the TME by METTL3-mediated m^6^A modification in CRC has been found. METTL3 silencing decreased the infiltration of MDSCs to sustain the activities of CD4^+^ and CD8^+^ T cells via the m^6^A-BHLHE41-CXCL1 axis. Furthermore, the combination of anti-PD1 therapy and METTL3 targeting led to synergistic antitumor efficacy in CRC.^228^ METTL3 was also reported to mediate the m^6^A methylation of the circRNA circIGF2BP3 (hsa_circ_0079587), boosting its circularization mediated via YTHDC1. Inhibition of circIGF2BP3 reduced the extent of immune escape and enhanced the anti-PD1 blockade immunotherapy response in a mouse model of Lewis lung cancer.^229^

**Fig. 5:**
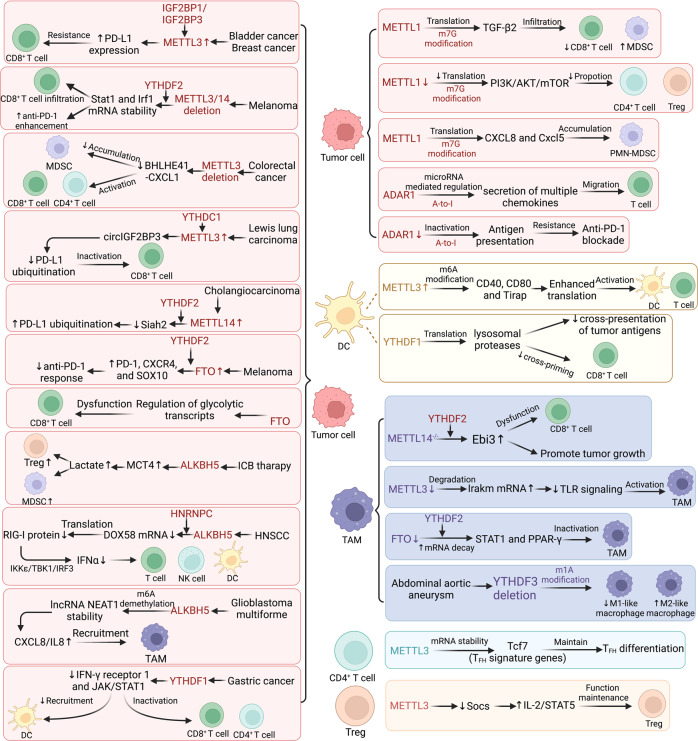
The main mechanisms by which RNA modification remodels the TME. Aberrant RNA modification of relevant genes in tumor cells has various effects in reprogramming the TME

In cholangiocarcinoma (CCA), METTL14 has been demonstrated to mediate the m^6^A modification of seven in absentia homolog 2 (Siah2), thereby regulating PD-L1 expression and modulating T-cell expansion and cytotoxicity.^230^ In addition, METTL3 and METTL14 have been found to be necessary for tumor growth and to play roles in the immune surveillance of senescent cells in mouse models.^231^ In cervical cancer (CC), METTL3 has been shown to be positively correlated with CD33^+^ MDSCs, which have been confirmed to exert suppressive roles in tumors and to be closely associated with poor prognosis in many patients with solid tumors.^232–234^ In esophageal squamous cell carcinoma (ESCC), increased METTL3 has been connected to a poor prognosis and has been found to be substantially correlated with the infiltration of effector memory CD8^+^ T cells, neutrophils, and NK cells.^235^ In HCC, an association between METTL3 and immune cell infiltration in the TME has also been reported. Shen and coworkers discovered that reduced METTL3 expression increased dendritic cells (DCs) infiltration and the expression levels of MHC molecules, adhesion molecules, and costimulatory molecules in HCC.^236^ Through CIBERSORT and survival analyses and gene set enrichment analysis (GSEA), METTL14 was found to be negatively correlated with Tregs and enrichment of chemokine-associated pathways. These above findings suggested that METTL14 could be a viable immunotherapy target in clear cell renal cell carcinoma (ccRCC).^237^ The roles of WTAP-modified tumor cells in remodeling the TME remain unclear. Recently, it was confirmed that WTAP, as another core component of the methyltransferase complex, is highly expressed in gastric cancer (GC) and is markedly correlated with T-cell infiltration in GC^238^ (Fig. 5).

All the aforementioned studies clarified the critical role of m^6^A methyltransferase-modified tumor cells in mediating tumor immune responses and remodeling the TME, suggesting novel strategies for improving immunotherapy.

*Roles of m*^*6*^*A erasers*: Yang et al. revealed that dysregulation of FTO expression in melanoma reduced the abundance of the m^6^A modification in the native protumorigenic genes PD1 (PDCD1), SOX10, and CXCR4 in melanoma cells and decreased RNA decay mediated through YTHDF2, ultimately leading to enhanced melanoma cell resistance to anti-PD1 blockade immunotherapy.^239^ FTO upregulates leukocyte immunoglobulin-like receptor B4 (LILRB4) expression in acute myeloid leukemia (AML), leading to immune response reprogramming. Small-molecule inhibitors of FTO sensitized leukemia cells to T-cell cytotoxicity and prevented immune system escape, which had been induced by hypomethylating agents.^240^

FTO has been demonstrated to upregulate the expression of c-Jun, JunB, and C/EBP, permitting reprogramming of the glycolytic metabolism. Targeting FTO with Dac51 rescued CD8^+^ T-cell function, and the combination of Dac51 and anti-PD-L1 blockade immunotherapy had a synergistic effect.^241^ These results highlight that targeting FTO is a potential cancer immunotherapy strategy.

Increasing evidence has revealed critical roles for ALKBH5, which regulates the TME in various types of cancer. A study of melanoma showed that ALKBH5 regulates the recruitment of tumor-infiltrating regulatory T cells (Tregs) and MDSCs by modulating the density of m6A marks and the number of splicing events during ICB therapy. Enhanced efficacy of immunotherapy has been observed after ALKBH5 inhibition.^242^ A unique mechanism through which ALKBH5 sustains PD-L1 expression in intrahepatic cholangiocarcinoma (ICC) in an m^6^A-dependent manner has been proposed to reduce MDSC infiltration and modulate immunotherapy efficacy.^243^ Recent findings revealed that ALKBH5 is also required for inhibiting the secretion of IFNα and reducing tumor cell infiltration in C3H immunocompetent mice via the m6A modification, providing a theoretical basis for targeting epitranscriptomic modulators in head and neck squamous cell carcinoma (HNSCC).^244^ In glioblastoma multiforme (GBM), deletion of ALKBH5 markedly repressed the recruitment of hypoxia-induced TAMs. CXCL8/IL8 expression and secretion were significantly reduced in ALKBH5-deficient tumors. ALKBH5 stabilized the lncRNA NEAT1 transcript after m^6^A demethylation in GBM, resulting in the upregulated secretion of CXCL8/IL8. Ectopic expression of CXCL8 induced the recruitment of TAMs and prevented the development of tumors caused by ALKBH5 inhibition. ALKBH5-mediated m^6^A modification induces TME remodeling under hypoxic conditions, suggesting a novel immunotherapeutic strategy for GBM.^245^ After performing GO and KEGG enrichment analyses, Wei et al. reported that ALKBH5-related genes showed enrichment in glioma immune signaling pathways. Further research validated the participation of ALKBH5 in the recruitment of M2 macrophages to glioma cells^246^ (Fig. 5).

*Roles of m*^*6*^*A readers*: YTHDF1 expression was reported to be significantly elevated in GC; deletion of YTHDF1 resulted in the recruitment of mature DCs as well as increased MHC class II expression and IL-12 secretion, resulting in the infiltration of CD4^+^ and CD8^+^ T cells with increased IFN- secretion. Bai confirmed the overexpression of YTHDF1 in GC and identified that YTHDF1 suppresses the DC-mediated antitumor immune response, indicating a potential role for YTHDF1 in GC treatment.^247^ In addition, YTHDF1 has been found to be significantly related to CD4-activated memory T cells, monocytes, macrophages, and activated NK cells in breast cancer. These findings indicate that YTHDF1 affects survival outcomes and immunotherapy responses in breast cancer.^248^ High expression of YTHDF1 and YTHDF2 was found to be significantly correlated with increased tumor-infiltrating lymphocyte (TIL) density in non-small cell lung cancer (NSCLC), indicating a potential role for these proteins in the TME.^249^

Lin et al. discovered that increased YTHDF2 expression was related to the poor overall survival of low-grade glioma (LGG) patients. YTHDF2 expression was also found to correlate with the invasion of immune cells into LGG, as evidenced by the upregulation of PD1, TIM-3, CTLA4, and TAM gene markers.^250^ In ccRCC, YTHDF2 expression has been confirmed to be markedly correlated with the abundance of immune cells, including CD8^+^ T cells, CD4^+^ T cells, macrophages, neutrophils, B cells, and DCs, indicating that it has potential as an indicator of ccRCC immune cell infiltration.^251^

Immune checkpoint gene expression and immune cell infiltration in lung adenocarcinoma (LUAD) are strongly correlated, and IGF2BP1 can serve as an independent predictor of LUAD prognosis and immunotherapy responses.^252^ Cui et al. observed that IGF2BP3 was markedly upregulated in BLCA, in which it regulated the membrane-bound and total PD-L1 expression.^253^

These findings indicate that the aberrant expression of m^6^A modulators in tumors usually affects the immune microenvironment in tumors, mediates immune escape, and ultimately leads to an immunosuppressive microenvironment. Thus, targeting m^6^A modification to prevent immune suppression and thereby restore the remodeling of the TME mediated via the m^6^A modification seems to be a promising strategy. However, the related research is still in the developmental stage. In-depth study into the mechanism of tumor m^6^A modification-induced remodeling of the TME and identification of other m^6^A-related regulatory factors will greatly enhance our understanding of the effects of the m^6^A modification on tumor immune regulation, leading to more effective antitumor therapy for cancer patients (Fig. 5).

##### N1-methyladenosine

The link between the m^1^A modification and the immune response in the TME has been the subject of an increasing number of investigations. Sun et al. performed a principal component analysis (PCA) and determined an m^1^A score based on the expression of 71 m^1^A-related genes and discovered that this score was strongly correlated with immunological features in colon cancer. The lower m^1^A score group exhibited effector CD8^+^ T proliferation, high PD-L1 expression and a superior anti-PD-L1 immunotherapy response, leading to prolonged survival and better prognosis. The study highlighted the importance of the m^1^A alteration in the remodeling of the TME. The m^1^A scoring system enables more effective characterization of immune cell infiltration, resulting in a more customized and successful antitumor immunotherapy strategy.^254^

Zhao et al. created a novel m^1^A-score model utilizing 10 m^1^A regulators and discovered that the m^1^A modification signature was associated with overall survival and the TME in HCC.^255^ An analysis of m^1^A methylation patterns in oral squamous cell carcinoma (OSCC) revealed a correlation between the m^1^A modification and TME characteristics. The results also showed that a high m^1^A score was closely linked to lower immune cell infiltration, lower expression of immune checkpoint molecules, and a poorer prognosis in OSCC.^256^ Similarly, m^1^A methylation exerts critical roles in predicting OC prognosis and remodeling the TME.^257^ These comprehensive m^1^A modification analyses have improved our understanding of the connection between m^1^A modification and immune cell infiltration, and provided a potential immunotherapy strategy. However, evidence confirming the importance of m^1^A modification in the TME is limited, most likely due to the difficulties in mapping this modification in the transcriptome and the scarcity of knowledge on the crucial roles of its regulation (Fig. 5).

##### 5-Methylcytosine

As the predominant methyltransferase of the m^5^C modification, increased NSUN2 expression in PCa has been correlated with poor clinical features. In addition, the expression of NSUN2 was found to be associated with the infiltration of multiple types of tumor cells, including memory B cells, resting memory CD4^+^ T cells, activating memory CD4^+^ T cells, and resting NK cells. Notably, elevated NSUN2 expression lowered PCa sensitivity to numerous chemotherapy drugs, implying that NSUN2 could be a potential therapeutic target for PCa.^258^ Tong et al discovered that NSUN2 expression was elevated in nasopharyngeal carcinoma (NPC), and that it was inversely linked with the infiltration of various immune cell. These results indicated that NSUN2 might be related to the immunotherapy sensitivity of NPC patients.^259^

Sylvain Delaunay and colleagues found that NSUN3-dependent m^5^C modification and the derivative f^5^C mark induced the translation of mt-mRNA, leading to increased metastasis. Deletion of NSUN3 in oral cancer cells failed to induce the invasion and dissemination of tumor cells.^260^ Pan et al. developed a prognostic risk signature for LUSC based on two m^5^C modulators, NSUN3 and NSUN4. NSUN3 and NSUN4 were found to be closely related to major immune cell infiltration. Among these modulators, NSUN3 was especially correlated with CD8^+^ T cells, while NSUN4 was associated with neutrophil infiltration.^261^

In pancreatic adenocarcinoma (PAAD), an m^5^C signature based on m^5^C regulators has been reported to be related to modulation of the TME and thus associated with tumor development. Moreover, the m^5^C score was determined to be associated with immune-related indicators that are thought to predict the immunotherapy efficacy in pancreatic ductal adenocarcinoma (PDAC).^262^ Using clinical and genetic transcriptome data of PDAC patients from the TCGA database, Yuan and colleagues developed a m^5^C-related lncRNA prognostic risk model. The risk model was found to be closely correlated with the TME, indicating a potential role for the m^5^C-related lncRNA prognostic risk model in the targeted treatment and prognosis of PDAC.^263^ In another study, the immunotherapy data associated with the m^5^C modification in 33 cancers was analyzed and it was found that NOP2 was elevated in most cancers and was closely correlated with tumor cell infiltration and immunotherapy efficacy. This comprehensive analysis provided evidence of the value of NOP2 in cancer immunotherapy, which deserves further research.^264^ In LUAD, it has also been found that m5C regulators associated with prognosis and immune cell infiltration.^265^ A comprehensive analysis of the TCGA database revealed that an m^5^C modification signature based on seven m^5^C regulators was significantly correlated with prostate adenocarcinoma biochemical recurrence and the diversity of the TME, providing new insights useful for the treatment of PCa^266^ (Fig. 5).

##### N7-methylguanosine

After radiofrequency ablation of recurrent HCC, METTL1 expression increased, which was accompanied by decreased CD8^+^ T-cell infiltration and increased infiltration of CD11b^+^ CD15^+^ polymorphonuclear myeloid-derived suppressor cells (PMN-MDSCs). The authors illustrated a novel mechanism by which METTL1 mediated the enhancement of TGF-β2 translation, significantly affecting the infiltration of PMN-MDSCs and CD8^+^ T cells. This study shed light on the critical role of METTL1 in altering TME and suggests a novel strategy for rescuing antitumor immunity.^267^ Another study showed that METTL1-mediated m^7^G modification enhanced SLUG/SNAIL translation under sublethal heat stress, indicating an essential role for the m^7^G modification in the recurrence of HCC after insufficient radiofrequency ablation (IRFA). Targeting the METTL1-m^7^G-SLUG/SNAIL axis may be beneficial for preventing HCC metastasis after IRFA.^268^ It was reported that METTL1-mediated tRNA m^7^G deposition regulates the PI3K/AKT/mTOR signaling pathway by modulating global mRNA translation. The pathway was verified to be involved in HNSCC. In addition, the results of single-cell RNA sequencing (scRNA‐seq) showed that METTL1 knockout markedly influenced immune cell infiltration. In Mettl1^cKO^ HNSCC, the proportions of Mrc1^+^ macrophages and Langerhans cells were significantly increased. Moreover, Mettl1^cKO^ HNSCC samples had a considerably lower proportions of exhausted CD4^+^ T cells and Tregs. These results indicated that METTL1 markedly alters the tumor immune landscape and may be a promising target in HNSCC patients.^269^ METTL1 has also been found to increase mRNA translation efficacy through increased recognition of codons within the mRNA translation process. In NPC cells, the METTL1/WNT/-catenin pathway promoted epithelial-mesenchymal transition (EMT) and chemoresistance. The aforementioned work demonstrated the crucial role of tRNA modification-mediated mRNA regulation in cancer progression.^270^ Very recently, it was reported that METTL1-mediated m^7^G modification significantly regulates PMN-MDSC accumulation in the TME and ICC progression through targeting CXCL8 in humans and Cxcl5 in mice. In preclinical animal models, blocking METTL1 and the downstream pathway together improved anti-PD1 therapy efficacy.^271^ All of the above studies reveal the crucial immunomodulatory function of m^7^G modification and provide potential clinical guidance.

An m^7^G-related lncRNA prognostic model based on TCGA data was analyzed to be related to immune cell infiltration. Similar results were observed in HCC,^272^ endometrial cancer,^273^ colon cancer,^274^ PCa^275^ and cutaneous melanoma.^276^ A comprehensive pancancer analysis found that METTL1 was strongly linked to tumor immune cell infiltration. Patients with a therapeutic response in the anti-PD-L1 group had greater METTL1 expression, providing novel guidance for tumor treatment.^277^ Yang and colleagues constructed an m^7^G score model based on 19 m^7^G methylation-related genes using the TCGA database and the Gene Expression Omnibus (GEO) database. The score of m^7^G was found to be correlated with tumor invasiveness, overall survival, ICI therapy responsiveness, and immune cell infiltration in PDAC patients. FN1 and ITGB1 were found to be key genes that inhibit the activation of T cells, resulting in immune evasion and diminished ICI therapy responses in PDAC.^278^ The correlation of m^7^G patterns with the TME in glioma has also been investigated in regard to immunological scores, immune cell infiltration, HLA, and immune checkpoint genes expression and immune-related functions. The high-risk group was shown to have increased infiltration of numerous immune cell types, including B cells; macrophages; immature DCs (iDCs); plasmacytoid DCs (pDCs); CD8^+^ T cells; neutrophils, T-helper cells, such as Th1 and Th2 cells; T follicular helper (TFH) cells; TILs; and Tregs.^279^ Similarly, an association of m^7^G modification with the TME in ccRCC has also been explored, and the m^7^G signature was found to be critical in the development of the TME in ccRCC. Therefore, the evaluation of the m^7^G modification has been beneficial in further guiding the treatment of ccRCC^280^ (Fig. 5).

##### Adenosine-to-inosine editing

ADAR enzyme-catalyzed A-to-I RNA editing has emerged as a major role in carcinogenesis and cancer progression. Naama Margolis and colleagues found that ADAR1 regulates the production of chemokines in melanoma, such as CCR4, CCR5, and CXCR3, in melanoma to recruit T lymphocytes. ADAR1 also regulates the release of several chemokines by melanoma cells. However, when T cells specifically identified melanoma cells based on their antigen expression, IFN-driven activation of ADAR1-p150 restored chemoattraction and boosted antigen-specific interactions. This positive feedback mechanism could have crucial effects on the growth of hot tumors as well as the great response to immunotherapy.^281^

Antienzyme inhibitor 1(AZIN1) is one of the most prevalent A-to-I RNA-edited proteins in several human cancers. The functional effects of RNA-edited AZIN1 on tumor angiogenesis have been exhaustively studied recently. RNA-edited AZIN1 has been reported to increase tumor angiogenesis by upregulating IL-8 in vivo and in vitro. Furthermore, the OAZ2-mediated ubiquitin-independent proteasome pathway was found to delay c-Myc degradation and enhance IL-8 secretion. These findings highlight the potential translational role of RNA-edited AZIN1 and highlight its significance in the vascular TME.^282^ ADAR1 has been reported to interact with Z-DNA-binding protein 1 (ZBP1) and to suppress ZBP1-mediated inflammatory cell death and apoptosis, leading to inhibition of antitumor immunity. These findings provide a new direction for leveraging A-to-I editing in tumor immunotherapy.^283^ It was discovered that ADAR1 deficiency destroyed cancer cells and reactivated immune-related pathways. In line with this, cancers with higher IFN-stimulated gene (ISG) profiles were discovered to be especially sensitive to ADAR1 knockdown, providing a theoretical basis for the treatment of tumors based on ISG signatures.^284^Some cancer types that are resistant to ADAR1 targeting can be made responsive by activating IFN signaling.^285^ According to Ishizuka et al., tumors became very sensitive to immunotherapy and ICI resistance was overcome after the RNA editing enzyme ADAR1 was disabled. In the absence of ADAR1, A-to-I editing of interferon-inducible RNA species was diminished, resulting in dsRNA ligand sensing by PKR and MDA5, which inhibited cell proliferation and promoted tumor inflammation, respectively. Inactivation of tumor cell antigen presentation eliminated the cellular resistance to PD1 checkpoint inhibition caused by loss of ADAR1.^286^

Recently, ADAR1 loss has been reported to trigger Z-form dsRNA (Z-RNA) element accumulation and activate Z-RNA sensor (ZBP1)-driven necroptosis. CBL0137 activates ZBP1-induced necroptosis, which can induce ADAR1 inhibition. In melanoma, CBL0137 can reverse the resistance to ICB therapy.^287^ These findings could reveal potential strategies for combating immunotherapy resistance and inhibiting ADAR1 may cause tumors to transition from an immunologically cold to an immunologically hot state (Fig. 5).

##### Pseudouridine

An efficient prediction model for predicting glioma prognosis was constructed using the Chinese Glioma Genome Atlas (CGGA) and TCGA datasets, and the model was applied to investigate the expression profiles of the Ψ synthase genes. The authors discovered that the risk score was significantly positively correlated with the malignant degree of glioma and the abundances of tumor-infiltrating immune cells (such as Tregs and M0 macrophages), but negatively correlated with the abundances of activated NK cells, monocytes and naive CD4^+^ T cells. The risk score was also found to be positively correlated with the expression of S100A11, CASP4, and other inflammatory markers in glioma. Overall, this work validated the role of the RNA Ψ modification in glioma malignancy and local immunity. In addition, it established the groundwork for future research of the relationship between Ψ and tumor immunity in tumors.^288^

##### N^4^-acetylcytosine

In an analysis of TCGA and GTEx data, NAT10 was found to be substantially expressed in most malignancies and significantly related to a poor prognosis. In addition, in HCC, NAT10 expression was considerably positively correlated with the immune infiltration of B cells, CD8^+^ T cells, CD4^+^ T cells, neutrophils, macrophages, DCs, endothelial cells, and fibroblasts, and strongly correlated with multiple immune-related marker gene sets.^289^ These findings suggest a potential regulatory function of ac^4^C in the tumor immune microenvironment, although ac^4^C mRNA alteration has been found to increase tumor cell proliferation and metastasis. However, it is not clear how ac^4^C RNA modification regulates the TME, and further investigation is required.

#### Chromatin remodeling

##### SWI/SNF

The SWI/SNF complex is repeatedly found to be mutated in cancer patients, and those with SWI/SNF mutations have been reported to be sensitive to ICIs. Nomogram analyses of 3416 patients in 6 reported cohorts showed that patients with ARID1A, ARID1B, and ARID2 mutations were more likely to benefit from ICI treatment.^290^

Shen et al. examined TCGA database and discovered that tumors with an increased TIL transcriptome profile had dramatically decreased ARID1A expression, regardless of ARID1A-associated enhancer activity.^291,292^ Notably, the researchers also found greater numbers of TILs and a notable increase in the CD8 protein cluster in tumors of syngeneic mice produced with an ARID1A-deficient ovarian cancer cell line compared to those in tumor of mice bearing an ARID1A-wild-type cell line.^292^ These findings corroborate the notion that ARID1A deficiency is associated with an increase in TILs, particularly CD8^+^ T cells, in the immune TME, suggesting that tumors harboring ARID1A loss are susceptible to immunotherapy.

PTEN inactivation is prevalent in PCa and is associated with a poorer prognosis.^293,294^ ARID1A, a subunit of the SWI/SNF chromatin-remodeling complex, has been shown to affect the immunosuppressive TME in PCa. The authors found that ARID1A deletion produced immunosuppressive TMEs in PTEN-deficient PCa and accelerated tumor progression. Inflammatory signals activate IKKβ to phosphorylate ARID1A, resulting in its destruction via β-TRCP. Inhibition of enhancers of A20 deubiquitinase, a critical negative regulator of NF-κB signaling, is a consequence of ARID1A downregulation. The IKK/ARID1A/NF-κB/CXCL-CXCR2 axis promotes PMN-MDSC recruitment to produce an immunosuppressive TME.^295^

Pbrm1 encodes components of the PBAF subunit of the SWI/SNF complex. Pan et al. discovered that the absence of PBAF function increases the sensitivity of melanoma cells to interferon-γ and increases the release of chemokines involved in effector T-cell recruitment. When Pbrm1 was inactivated, immunotherapy responsiveness was increased.^296^ Similar results have also been reported in rhabdoid tumors (RTs). PBRM1 levels are inversely correlated with CD8^+^ cytotoxic T-cell infiltration, which suggests immunotherapeutic potential in RTs.^297^ ARID2 mutations are significantly more prevalent than PBRM1 mutations in melanoma. Therefore, the role of ARID2 as a tumor immunomodulator is further studied. ARID2 silencing leads to upregulation of signal transducers and activators of transcription 1 (STAT1), which subsequently leads to increased expression of CXCL9, CXCL10, and CCL5. Knockout of ARID2 sensitizes melanoma to ICIs with increased infiltration of cytotoxic CD8^+^ T cells.^298^

SMARCA4, the key ATPase component of the SWI/SNF chromatin-remodeling complex, controls transcription via the regulation of chromatin structure and is increasingly believed to play a substantial role in human malignancies. A comprehensive analysis of SMARCA4 revealed that it is significantly expressed in numerous types of cancer and is related with poor overall survival in some tumors. SMARCA4 is related to several immune cells and genes in various forms of cancer. SMARCA4 dysregulation is associated with tumor mutation burden (TMB), mismatch repair (MMR), microsatellite instability (MSI) and DNA methylation. The expression of SMARCA4 is low in esophageal carcinoma (ESCA), prostate adenocarcinoma (PRAD), and skin cutaneous melanoma (SKCM). These findings provide a thorough understanding of the carcinogenic consequences of SMARCA4 in various cancers, which may be linked to tumor immunity in various cancers.^299^

##### CHD

A recent study provided evidence for an important role of CHD1 in Pten-deficient PCa. In genetically engineered Pten and Pten/Smad4 animal models, deletion of Chd1 dramatically inhibits tumor growth and extends survival. IL-6, a crucial target of CHD1 transcription, plays a key role in MDSC recruitment. In Pten-deficient PCa, Chd1 deletion remodels the TME, reduces MDSC recruitment, and increases CD8^+^ T-cell infiltration. Pharmacological inhibition of IL-6 combined with ICB in PCa elicits a powerful antitumor response.^300^

As a crucial component of the ATP-dependent nucleosome remodeling and deacetylase (NuRD) complex, metastasis-associated protein 1 (MTA1) is frequently overexpressed in cancers. In CRC, upregulation of MTA1 expression induces an immunosuppressive TME. Upregulation of mta1 promotes tumor progression by reducing tumor macrophages, causing residual macrophages to transition into a TAM phenotype, and blocking the activation of cytotoxic T lymphocytes (CTLs), thereby forming an immunosuppressive TME.^301^

Given the diverse and significant impact of chromatin remodeling on the immune TME, additional research is required. As immunosuppressive TME is regarded as the primary cause of immunotherapy resistance, these findings will be beneficial for identifying novel targets to increase the efficacy of immunotherapy.

#### Noncoding RNAs

With technological progress, the function of ncRNAs in tumors has been intensively investigated. Intercellular communication within the TME is essential for tumor progression. Numerous studies have demonstrated that exosomes contain an abundance of ncRNAs.

HCC cells produce high levels of the exosomal lncRNA TUC339. This lncRNA promotes M2 polarization, which in turn reduces the production of proinflammatory cytokines, hinders phagocytosis, and decreases the expression of costimulatory molecules in macrophages.^302^ miR-21-5p expression is very abundant in CRC cell exosomes and promotes M1 polarization via TLR7 and the release of IL-6, establishing a proinflammatory pre-metastatic microenvironment and ultimately leading to liver metastasis.^303^ Recent research shows that epigenetically inhibiting the miR-144/451a cluster epigenetically promotes HCC development via paracrine HGF/MIF-mediated TAM remodeling.

Increased M2 polarization, cancer cell migration, and invasion result from exposure to exosomal miR-21 from tumor cells. This miR-21 regulates PI3K/AKT signaling by downregulating PTEN activation in macrophages and upregulating STAT3 expression.^304^ Similar results were also observed for exosomal miR-130b-3p, miR-425-5p, and miR-25-3p.^305^ In addition, exosomal circFARSA induced M2 polarization by activating PI3K/AKT signaling in macrophages via ubiquitination and degradation of PTEN.^306^ In HCC, circUHRF1 is secreted by HCC cells in an exosomal manner and inhibits the secretion of IFN-γ and TNF-α derived from NK cells, thereby inhibiting NK cell function and possibly driving resistance to anti-PD1 immunotherapy.^307^

CircARSP91 participates in tumor immune surveillance by elevating UL16-binding protein 1 (ULBP1) mRNA and protein expression to promote NK cell function and by elevating the NK-mediated immune response in HCC.^308^ The circ-0000977/miR-153/HIF1 axis suppresses NK cell death, which contributes to HIF1-mediated immunological escape of PCa cells.^309^ These results indicate the influence of noncoding RNA on the TME and the potential of targeting them to improve antitumor immunotherapy.

### Epigenetic modifications of immune cells in the TME

#### Epigenetic modulation of DCs

DCs have a crucial role in regulating the adaptive and innate immune responses. To regulate T-cell development, they integrate signals from pathogens or other damage signals and present processed antigens to naive T cells.^310–312^

##### Histone modification

Forkhead box transcription factor M1 (FOXM1) has been reported to participate in oncogenesis by transcriptionally regulating of target genes in several cells, including DCs.^313–315^ Recent studies have shown that FOXM1 delays the maturation of bone marrow-derived dendritic cells (BMDCs) and inhibits T-cell proliferation in tumor-bearing mice. Mechanistically, the enrichment of H3K79me2 in the FOXM1 promoter was observed, and FOXM1 expression was regulated by epigenetic inheritance. Inhibition of FOXM1 expression was repressed by the H3K79 methyltransferase DOT1L, which partially reversed its immunosuppressive effect on BMDCs. These results suggest that the H3K79me2/FOXM1/Wnt5a pathway significantly inhibits the maturation phenotype and effects of BMDCs in colon and pancreatic cancer^313^ (Fig. 4).

##### RNA modification

METTL3 deletion in DCs impairs the phenotypic and functional maturation, leading to downregulated expression levels of CD40, CD80, and IL-12. Regarding the underlying mechanism, METTL3-mediated m^6^A modification of CD40, CD80, and Tirap enhances their translation in DCs, further stimulating the activation of T cells, revealing a novel mechanism of m^6^A modification-mediated DC activation and T-cell response.^316^ Wu and colleagues observed similar results, with the depletion of METTL3 in DCs reducing the levels of MHCII, CD80, CD86, IL-12, and IFN-γ and impairing T-cell activation.^317^

YTHDF1 augments the translational efficacy of cathepsin transcripts in classic DCs, thus promoting lysosomal protease expression and leading to limited immune recognition. Deletion of YTHDF1 in DCs enhances the ability of DCs to cross-prime CD8^+^ T cells, which suggests that YTHDF1 reduced the ability of DCs to present tumor neoantigens to T cells. These results highlight the critical role of YTHDF1 in mediating immune evasion, and therefore, agents targeting YTHDF1 will likely have synergistic effects with ICB treatment^318^ (Fig. 5).

##### Noncoding RNA

By cross-priming CD8 + T cells, DCs in the TME play a critical role in both the induction and maintenance of antitumor T-cell immunity.^312^ By binding directly to the C-terminus of STAT3, Lnc-DC prevents SHP1 from dephosphorylating STAT3, which speeds up the phosphorylation of tyrosine-705 of STAT3 and the expression of genes implicated in DC activation. Knockdown of lnc-DC inhibits DC differentiation from human monocytes, decreases their ability to promote T-cell activation, and decreases the expression of function-related genes and antigen absorption.^319^ LncRNA HOTAIRM1 is also involved in the DC differentiation.^320^ However, further research is needed to discover the function of these lncRNAs within DCs during malignant transformation.

#### Epigenetic modulation of MDSCs

MDSCs are immature cells of myeloid origin and have an exceptional capacity to inhibit T-cell responses.^321^ In addition to their suppressive effects on adaptive immune responses, MDSCs modulate the cytokine production of macrophages to regulate innate immunological responses.^322^

##### DNA methylation

The accumulation of MDSCs is a hallmark of cancer, though the mechanisms causing MDSC growth in the TME remain unknown. Alyssa D. Smith discovered that inhibiting DNMTs using the DNA methyltransferase inhibitor decitabine (DAC) reduced the formation of MDSCs and accelerated the activation of antigen-specific cytotoxic T cells. The authors found that MDSCs limit TNFα expression via a STAT3-DNMT epigenetic axis controlled by autocrine IL-6. This reduces the necrosis induced by TNFα and causes a reliance on RIP1 for survival and accumulation. Thus, reducing IL-6 expression or function may be a potentially beneficial strategy for decreasing MDSC survival and accumulation in the TME^323^ (Fig. 3).

##### Histone modification

MDSCs play a central role in tumor immune escape and tumor metastasis and are negatively associated with prognosis and survival in cancer patients. Recently, Varun Sasidharan Nair and colleagues analyzed different subpopulations of MDSCs in cancer: monocytic MDSCs (M-MDSCs), immature MDSCs (I-MDSCs), and polymorphonuclear/granulocyte MDSCs (PMN-MDSCs). The results of the investigation revealed that the levels of I-MDSCs and PMN-MDSCs were higher in CRC tumor tissues than in normal tissues. In tumor-infiltrating I-MDSCs, genes associated with HDAC activation and DNA methylation-mediated transcriptional silencing were upregulated, whereas genes associated with HATs were downregulated. Notably, PMN-MDSCs showed dysregulation of genes associated with DNA methylation and HDAC binding. In vitro, a reduction in HDAC activity in CRC tumor tissue decreased immunosuppression and myeloid chemotaxis-related gene expression, supporting the significance of HDAC activation in MDSC functions and chemotaxis^324^ (Fig. 4).

##### Noncoding RNAs

Several lncRNAs, including lnc-CHOP and lnc-C/EBP, have been identified to control the generation, recruitment, and immunosuppressive effects of MDSCs. Lnc-CHOP interacts with CHOP and C/EBP isoform liver-enriched inhibitory protein (LIP) to enhance the activation of C/EBP, leading to the production of key molecules associated with MDSC immunosuppressive function, such as ARG1 and NOS2.^325^ A recent study showed that the immunosuppressive function and differentiation of M-MDSCs was stimulated in vitro and in vivo by means of a regulatory network composed of m6A-modified Olfr29-ps1/miR-214-3p/MyD88.^326^

#### Epigenetic modulation of TAMs

TAMs constitute the majority of immune cells in the TME. They play a significant role in tumor formation, invasion, metastasis, immunosuppression, angiogenesis, and drug tolerance by secreting cytokines and chemokines and coordinating with inflammatory processes.^327–329^

##### RNA modification

Deletion of METTL14 in C1q^+^ TAMs downregulated m^6^A abundance and increased Epstein‒Barr virus-induced protein 3 (EBI3) levels, which shifted the CD8^+^ T cells toward dysfunctional state. Recently, METTL3 deletion was reported to increase the M1/M2-like TAM ratio and augment the infiltration of Tregs into tumors. Moreover, METTL3-deficient mice showed attenuated therapeutic efficacy of an anti-PD1 blockade, implying that METTL3 could be a potential target in tumor immunotherapy.^219^ Tong et al. confirmed that macrophages lacking METTL3 produced less TNF-α. METTL3 facilitated the m^6^A modification of the IRAKM mRNA, which encodes a negative TLR4 signaling regulator. Hence, loss of METTL3 slowed IRAKM degradation, leading to higher IRAKM expression and impaired TLR signaling-mediated macrophage activation.^330^

METTL3 has also been found to direct the m^6^A methylation of JAK1 mRNA in tumor-infiltrating myeloid cells (TIMs), and the m^6^A/YTHDF1 axis has been shown to promote JAK1 translation. In addition, lactylation increased METTL3 expression in TIMs. These findings highlighted the contribution of lactylation-driven METTL3-mediated m^6^A methylation to the immunosuppressive effects of TIMs.^331^ METTL3 has also been observed to be necessary for M1 macrophage polarization because it induces m^6^A methylation of STAT1.^332^

FTO silencing accelerated the mRNA decay of STAT1 and PPAR-γ in a YTHDF2-dependent manner, thus impeding the activation of macrophages. This study shed new light on the relationship between FTO and macrophage polarization.^333^ In contrast to the positive roles played by m^6^A writers in macrophages, YTHDF2 knockdown in macrophages (RAW264.7 cells) enhanced the expression of LPS-induced inflammatory cytokines, such as IL-1β, IL-6, TNF-α, and IL-12, resulting in an increasingly severe inflammatory response.^334^ Wu and colleagues found that the m1A reader YTHDF3 was highly expressed in abdominal aortic aneurysm (AAA) and located in macrophages, as determined by immunofluorescence staining of the AAA adventitia. Macrophage M1 polarization was decreased while macrophage M2 polarization was increased when YTHDF3 was deleted from M0 macrophages. These findings suggest a unique mechanism by which the m^1^A modification may be essential for macrophage polarization and regulation of the TME^335^ (Fig. 5).

##### Noncoding RNA

In GBM, the lncRNACASC2c binds to coagulation factor X (FX) and reduces its synthesis and secretion, hence limiting macrophage movement and polarization to the M2 subtype. Tumor microenvironmental FX suppression is critical for M2 macrophage polarization because it reduces macrophage ERK1/2 and AKT phosphorylation and activation.^336^ Paracrine activation of Wnt/β-catenin signaling in macrophages is achieved by LINC00662 through upregulation of WNT3A expression and secretion in HCC. Therefore, LINC00662 enhances M2 macrophage polarization, resulting in the growth and spread of HCC tumors. High LINC00662 expression in HCC is associated with overactivated WNT3A, M2 macrophage polarization, and poor prognosis in HCC patients, according to additional clinical evidence.^337^ Targeting these lncRNAs in the TAMs or tumor cells could be a feasible antitumor therapeutic strategy because they together alter the functions of TAMs via many mechanisms and influence carcinogenesis and metastasis.

#### Epigenetic modulation of tumor-infiltrating lymphocytes (TILs)

##### DNA methylation

The poor differentiation of DNMT1-deficient lymphocytes highlights the importance of DNA methylation in CD8^+^ T cells, but enhanced immunological memory responses are seen in Tet2-/-lymphocytes.^338,339^ Increased DNMT1 synthesis by CD8^+^ T cells in the TME is correlated with increased methylation of genes linked with T-cell dysfunction, which in turn suppresses the antitumor phenotype of these cells.^340^ To counteract the negative effects of de novo epigenetic programming on PD1 blockade-mediated T-cell rejuvenation, DNMT inhibitors may boost T-cell rejuvenation and enhance antitumor activity in response to anti-PD1 therapy.^341^

In the TME, regulatory T cells (Tregs), an immunosuppressive subgroup of CD4^+^ T cells characterized by the expression of the master transcription factor Forkhead box protein P3 (FOXP3), accumulate and may even make up the vast majority of invading CD4^+^ T cells.^342^ Tet1 and Tet2 catalyze the conversion of 5mC to 5hmC in Foxp3, generating a hypomethylation pattern unique to Tregs and sustaining Foxp3 expression, according to Yang et al. Therefore, loss of Tet1 and Tet2 results in hypermethylation of Foxp3 and reduced differentiation and function of Tregs, providing a theoretical foundation for investigating epigenetic alterations and tumor immune microenvironment homeostasis^343^ (Fig. 3).

##### Histone modification

Significantly increased expression of serine/arginine-rich splicing factor 2 (SRSF2) was observed in exhausted T cells. Mechanistically, SRSF2 was found to regulate the transcription of these genes by binding to the acyltransferase P300/CBP complex and altering H3K27Ac levels near the immune checkpoint molecules, ultimately leading to the recruitment of STAT3 to these gene promoters. These results not only indicate that SRSF2 has the potential to be a target for reversing TIL exhaustion, but also illustrate the role of histone modification in the TME, providing more abundant evidence in this field.^344^

The hypofunctional differentiation state of exhausted T cells is partly responsible for the low response rates to immunotherapy in solid tumors. By analyzing the histone modification landscape of tumor-infiltrating CD8^+^ T cells during differentiation, Ford et al. found that the reduction in the transcriptional potential of terminally exhausted T cells was driven by increased histone bivalence, which was correlated with hypoxic exposure. Increasing the level of the hypoxia-insensitive histone demethylase Kdm6b promoted the antitumor immunity. The aforementioned findings indicate that certain epigenetic modifications mediated by histone modifications during T-cell development promote exhaustion. This raises the possibility that the transcriptional potential of terminally exhausted T cells can be restored in the presence of increased costimulatory signals and reduced hypoxia.^345^

The capacity of TH1 cells to stimulate CD8^+^ antitumor T-cell responses is often associated with an active antitumor immune response.^346^ Recent studies have highlighted the role of epigenetic inheritance in promoting the differentiation of specific T-helper cell lineages. Loss of EZH2 and PRC2 activity promotes both TH1 and TH2 cell accumulation,^347^ while the SUV39H1–H3K9me3–HP1 pathway promotes TH2 cell development by silencing TH1 cell-related genes.^348^ In addition, SETDB1-dependent H3K9me3 suppresses the expression of genes related to TH1 cells in CD4^+^ cells, leading to the control of TH1 cells in vitro^349^ (Fig. 4).

##### RNA modification

Deletion of METTL3 in mouse T cells was reported to disrupt homeostatic expansion of naive T cells. In naive METTL-deficient T cells, the SOCS1, SOCS3, and CISH expression was increased in an m^6^A-dependent manner, thereby blocking IL-7-mediated activation of STAT5 and reprogramming T-cell homeostasis and differentiation. This was the first study to illustrate the critical role of the m^6^A modification in T-cell homeostasis and differentiation, highlighting a unique T-cell regulatory mechanism.^350^ TFH cells are CD4^+^ T cells that play a crucial role in humoral immunity.^351^ Yao et al. discovered that deleting METTL3 from CD4^+^ T cells in mice inhibited TFH cell development and maturation by modifying Tcf7 mRNA via m^6^A modification. These findings suggest that Tcf7 mRNA stability is regulated via METTL3, indicating pivotal roles for the m^6^A modification in promoting TFH cell differentiation.^352^ Further research revealed that depletion of METTL3 in Tregs boosted Socs family member expression, leading to the suppression of the IL-2/STAT5 signaling pathway, which is essential for Treg function and stability. Since Tregs play roles in tumor immunosuppression, selective targeting of m^6^A in Tregs is expected to benefit cancer immunotherapy.^353^

During autoimmunity, ALKBH5 increases the m^6^A modification of IFN-γ and C-X-C motif chemokine ligand 2 (CXCL2), leading to decreased mRNA stability and protein levels in CD4^+^ T cells, according to a recent study. This finding demonstrated that ALKBH5 functions in regulating CD4^+^ T cells effects in autoimmune responses.^354^ Recent bioinformatics analysis found that PCa patients with high HNRNPC expression exhibit immunosuppressive TME with higher Treg infiltration and suppressed effector CD8^+^ T cells. Targeting HNRNPC may be a potential therapeutic strategy for advanced PCa.^355^ (Fig. 5).

##### Noncoding RNA

A negative correlation exists between the upregulated expression of lnc-Tim3 in HCC patients and the production of IFN-γ and IL-2 by tumor-infiltrating CD8^+^ T cells.^356^ Similarly, lnc-sox5 is significantly elevated in CRC, and lnc-sox5 knockout significantly promotes CD8^+^ T infiltration and cytotoxicity by inhibiting indoleamine 2, 3-dioxygenase 1 (IDO1) expression, thereby inhibiting CRC tumorigenicity.^357^

In patients with HCC, lnc-EGFR is highly expressed in Tregs, where it binds specifically to EGFR, prevents its ubiquitination and degradation, and maintains the downstream activation of AP-1 and NF-AT1 (two transcription factors of FOXP3), thereby enhancing the immunosuppressive function of Tregs and promoting HCC progression.^358^ It has been revealed that the Treg lncRNAs Flicr and Flatr, both of which are highly conserved and enriched in activated Tregs, control FOXP3 expression and the immunosuppressive function of Tregs.^359,360^

#### Epigenetic modulation of NK cells

NK cells are innate immune system cytotoxic lymphocytes that kill their targets and release cytokines. They regulate other immune cells and are responsible for controlling viral and intracellular bacterial infections and cancers.^361–363^

##### RNA modification

As a crucial component of tumor immune surveillance, NK cells are innate lymphoid immune cells with the capacity to specifically target and eradicate cancer cells.^361,364^ Song and colleagues reported that deletion of METTL3 in NK cells downregulated protein expression of SHP-2 in a manner mediated by m^6^A modification, thereby rendering NK cells hyporesponsive to IL-15 and leading to reduced NK cell infiltration and dysfunction. These findings indicated that METTL3-mediated m^6^A modification maintains the tumor immunosurveillance of NK cells^365^ (Fig. 5).

##### Noncoding RNA

The most commonly researched lncRNA in NK cells is lnc-CD56, which regulates CD56, a classic human NK surface marker. Knockdown of lnc-CD56 lowers CD56 expression, suggesting that lnc-CD56 is a positive regulator of CD56 and is required for the development and various functions of NK cells.^366^ LncRNA GAS5 is downregulated in NK cells of liver cancer patients, although it is upregulated in activated NK cells relative to non-stimulated NK cells. By regulating miR-544/RUNX3, overexpression of GAS5 in activated NK cells increases IFN-γ production, NK cytotoxicity, and the proportion of CD107a^+^ NK cells, hence boosting the killing actions of NK cells and suppressing tumor growth.^367^ These findings emphasize the significance of NK cell activities and antitumor immunity.

### Targeting epigenetics for cancer immunotherapy

#### Epigenetic drugs

In the last few years, intensive efforts have been devoted to epigenetic targeting. Up to now, many epi-drugs have been FDA-approved or under clinical trials for the treatment of both hematological and solid tumors (Table 1). Combining epigenetic targeting agents with immune checkpoint inhibitors (anti-PD1, anti-PD-L1, etc.) is currently being evaluated in numerous clinical studies due to the synergistic effects of the combination of the two treatments^368–371^ (Table 2).

**Table 1:** Epigenetic drugs approved or under clinical trials

Epigenetic targeting	Category	Approved drugs and conditions	Drugs under clinical trials
DNA methylation	DNMT inhibitor	Azacitidine (juvenile myelomonocytic leukemia/acute myeloid leukemia), Decitabine (myelodysplastic syndromes)	Guadecitabine, Tioguanine, FdCyd, TdCyd, aza-TdC, RX-3117, NTX-301
Histone modification	HDAC inhibitor	None	Vorinostat, Romidepsin, Panobinostat, Belinostat, Valproic acid, Chidamide, Mocetinostat, Entinostat, Abexinostat, Domatinostat, Tinostamustine, ACY-241, KA-2507, AR-42, Nanatinostat, ITF2357, SB939, Resminostat, ACY-1215
	BET inhibitor	None	INCB057643, BMS-986158, JAB-8263
	EZH2 inhibitor	Tazemetostat (advanced epithelioid sarcoma/follicular lymphoma)	CPI-1205, PF-06821497, SHR2554, CPI-0209
	KDM1A inhibitor	None	Seclidemstat, IMG-7289, GSK2879552, INCB059872

**Table 2:** Clinical trials combining epigenetic targeting agents and immune checkpoint inhibitors

Epigenetic targeting agents	Immune checkpoint inhibitors	Conditions and trial ID
DNMTi		
Azacytidine	Pembrolizumab (anti-PD1)	CRC (NCT02260440, NCT02512172), NSCLC, microsatellite-stable CRC, HNSCC, urothelial carcinoma and melanoma (NCT02959437), AML (NCT02845297, NCT04284787, NCT03769532), NSCLC (NCT02546986), myelodysplastic syndrome (MDS) (NCT03094637), melanoma (NCT02816021), PDAC (NCT03264404), platinum-resistant ovarian cancer (NCT02900560), relapsed/refractory Hodgkin lymphoma (NCT05355051)
	Nivolumab (anti-PD1)	AML (NCT02397720, NCT03825367, NCT04913922), NSCLC (NCT01928576), MDS (NCT02530463) osteosarcoma (NCT03628209), hodgkin lymphoma (NCT05162976), HNSCC (NCT05317000)
	Durvalumab (anti-PD-L1)	MDS (NCT02281084, NCT02117219, NCT02775903), AML (NCT02775903), peripheral T-cell lymphoma (NCT03161223), NSCLC (NCT02250326), pancreatic cancer (NCT04257448), microsatellite-stable CRC, ovarian cancer and estrogen receptor-positive and HER2-negative breast cancer (NCT02811497)
	Camrelizumab (anti-PD1)	AML (NCT05772273), peripheral T-Cell lymphoma (NCT05559008)
	Atezolizumab (anti-PD-L1)	MDS (NCT02508870)
	Avelumab (anti-PD-L1)	AML (NCT03390296)
	Spartalizumab (anti-PD1)	MDS/AML (NCT03066648)
	Tremelimumab (anti-CTLA4)	MDS (NCT02117219)
	Ipilimumab (anti-CTLA4)	MDS (NCT02530463), AML(NCT02397720)
	PF-04518600 (anti-OX40)	AML (NCT03390296)
Decitabine	Pembrolizumab	AML (NCT02996474, NCT03969446), MDS (NCT03969446), peripheral T-cell lymphoma/cutaneous T-cell lymphoma (NCT03240211), CNS solid tumors and lymphomas (NCT03445858), NSCLC (NCT03233724), locally advanced HER2-negative breast cancer (NCT02957968), metastatic triple-negative breast cancer (NCT05673200)
	Nivolumab	Mucosal melanoma (NCT05089370), AML (NCT04277442), NSCLC (NCT02664181), MDS/AML (NCT02664181)
	Durvalumab	head and neck cancer (NCT03019003),
	Camrelizumab	Hodgkin lymphoma (NCT04510610, NCT03250962, NCT04514081, NCT04233294), primary mediastinal large B-cell lymphoma (NCT03346642), AML(NCT04353479)
	Spartalizumab	MDS (NCT05201066), MDS/AML (NCT03066648)
	Ipilimumab	Relapsed or refractory myelodysplastic syndrome or AML (NCT02890329)
Guadecitabine	Pembrolizumab	Lung cancer (NCT03220477), NSCLC/castration-resistant prostatic cancer (NCT02998567), ovarian, primary peritoneal or fallopian tube cancer (NCT02901899)
	Nivolumab	Melanoma, NSCLC (NCT04250246)
	Durvalumab	Advanced kidney cancer (NCT03308396), extensive-stage small cell lung cancer (NCT03085849), hepatocellular carcinoma, gallbladder cancer, pancreatic cancer, intrahepatic cholangiocarcinoma (NCT03257761)
	Atezolizumab	Chronic myelomonocytic leukemia, MDS and AML (NCT02935361), AML (NCT02892318), ovarian, fallopian tube, or primary peritoneal cancer (NCT03206047), urothelial carcinoma (NCT03179943)
	Tremelimumab	Extensive-stage small cell lung cancer (NCT03085849)
	Ipilimumab	Melanoma (NCT02608437), melanoma/NSCLC (NCT04250246)
HDACi		
Vorinostat	Pembrolizumab	HNSCC or salivary gland cancer (NCT02538510), NSCLC (NCT02638090), renal or urothelial cell carcinoma (NCT02619253), glioblastoma (NCT03426891), diffuse large B-cell lymphoma, follicular lymphoma or Hodgkin lymphoma (NCT03150329), squamous cell carcinoma (NCT04357873), breast cancer (NCT04190056)
Entinostat	Pembrolizumab	MDS (NCT02936752), advanced solid tumors (NCT02909452), lymphoma (NCT03179930), NSCLC, melanoma and CRC (NCT02437136), melanoma (NCT03765229), uveal melanoma (NCT02697630), bladder cancer (NCT03978624)
	Nivolumab	CNS tumor, solid tumor (NCT03838042), renal cell carcinoma (NCT03552380), HER2-negative breast cancer (NCT02453620), non-small cell lung cancer (NCT01928576), cholangiocarcinoma or PDAC (NCT03250273)
	Ipilimumab	HER2-negative breast cancer (NCT02453620), renal cell carcinoma (NCT03552380)
	Atezolizumab	Triple-negative breast cancer (NCT02708680), lung cancer (NCT04631029), hormone receptor-positive, HER2-negative breast cancer (NCT03280563), renal cell carcinoma (NCT03024437)
	Avelumab	Ovarian, peritoneal and fallopian tube cancer (NCT02915523)
Panobinostat	Ipilimumab	Melanoma (NCT02032810)
	Nivolumab	Triple-negative breast cancer (NCT02393794)
	Durvalumab	Lymphoma (NCT03161223)
Mocetinostat	Pembrolizumab	Lung cancer (NCT03220477)
	Nivolumab	NSCLC (NCT02954991)
ACY-241	Nivolumab	NSCLC (NCT02635061), Melanoma (NCT02935790)
	Ipilimumab	Melanoma (NCT02935790)
Valproic acid	Avelumab	Virus-associated cancers (NCT03357757)
inostamustine	Nivolumab	Advanced melanoma (NCT03903458)
Chidamide	Nivolumab	Melanoma, renal cell carcinoma, NSCLC (NCT02718066), advanced melanoma (NCT04674683)
	Pembrolizumab	NSCLC (NCT05141357)
Abexinostat	Pembrolizumab	Solid tumors (NCT03590054)
Domatinostat	Avelumab	Merkel cell carcinoma (NCT04393753), Gastrointestinal cancers (NCT03812796)
	Nivolumab and ipilimumab	Melanoma (NCT04133948)
Nanatinostat	Pembrolizumab	EBV-positive solid tumors (NCT05166577)
BET inhibitor		
BMS-986158	Nivolumab	Selected advanced tumors (NCT02419417)
EZH2 inhibitor		
Tazemetostat	Pembrolizumab	Urothelial carcinoma (NCT03854474), HNSCC (NCT04624113)
	Atezolizumab	Follicular lymphoma and diffuse large B-cell lymphoma (NCT02220842)
	Nivolumab and ipilimumab	Malignant rhabdoid tumor, atypical teratoid rhabdoid tumor, epithelioid sarcoma, and chordoma (NCT05407441)
	Durvalumab	Advanced solid tumors (NCT04705818)
CPI-1205	Ipilimumab	Advanced solid tumors (NCT03525795)
KDM1A inhibitor		
IMG-7289	Atezolizumab	Extensive-stage small cell lung cancer (NCT05191797)

#### DNA methylation

Reprogramming M2-type TAMs into M1-type is a critical strategy for tumor therapy. NK cells treated with 5-azacytidine (5-aza, a DNA methyltransferase inhibitor) exhibit improved effector capabilities, suggesting additional potential for epigenetic targeting.^362^ A recent study has shown that, in comparison to either therapy alone, combination of the DNA methylation inhibitor 5-aza-2’-deoxycytidine (5-aza-dC) and the histone deacetylation inhibitor trocomycin A (TSA) lowers cytokine levels in M2-type macrophages and the cytokine levels of M1-type macrophages. In addition, treatment of 5-aza-dC and TSA in the conditioned medium of M2 macrophages sensitized tumor cells to paclitaxel.^372^

Few functional investigations have determined the epigenetic dependencies of T-helper cells. However, the effect of epigenetic modulators on Tregs has been well-defined. There is interest in using these epigenetic mechanisms therapeutically to eliminate Treg cell-mediated immunosuppression in cancer, as Tregs are among the most important immunosuppressive cells in the TME.^373,374^ One of the most significant locations in the DNA methylation profile of Tregs is the FOXP3 gene itself.^375,376^ The FOXP3 gene contains at least three conserved noncoding sequences (CNS1–CNS3) in cis, the methylation state of which determines FOXP3 expression and stability.^377^ In human CD4^+^ T cells, methylation of the core FOXP3 promoter inversely correlates with FOXP3 expression, which increases during coculture with tumor cells.^378^ Although 5-aza treatment boosts FOXP3 expression initially, it ultimately leads to loss of suppressive activity and increased production of proinflammatory cytokines. This is presumably the outcome of global epigenetic remodeling beyond the Treg-specific demethylation zone (TSDR).^379,380^ These findings provide additional mechanistic support for the use of hypomethylating drugs to enhance immunotherapy based on checkpoint inhibitors.

Epigenetic combination therapy showed a robust antitumor response and survival benefit. Compared with either treatment alone, combination of ADAR1 deletion and DNMTi was found to significantly increase proinflammatory cytokine production and IFN-β sensitivity. The combination remodels the TME through enhancing the activation and recruitment of CD8^+^ T cells and reduces the tumor burden in the OC mouse model.^381^ Combining anti-PD-L1, anti-PD1, or anti-CTLA4 therapy with a DNMT inhibitor enhances the antitumor immune response of CD8^+^ T cells and promotes a type I interferon response; clinical trials combining methyltransferase inhibitors and ICIs have begun.^341,382–384^ Utilizing hypomethylating drugs to revitalize CD8^+^ T cells prior to immune checkpoint therapy could be a useful strategy.

##### Histone modification

As mentioned previously, epigenetic modification plays a role in the regulation of T-helper cell differentiation. Thus, new SUV39H1 or HP1 inhibitors may be employed in cancer immunotherapy to stimulate TH1 cell activity and improve antitumor immune responses. However, this utility has not been experimentally validated.^348^ Given the role of SETDB1 in regulating T-helper cell lineage integrity (described above), these findings indicate the possibility for inhibitors of selective epigenetic modifications to improve antitumor immunity via different pathways.

In addition, histone modification is also a critical determinant of Treg development and function. Systemic delivery of a class-I/II HDAC inhibitor (trichostatin A) to mice resulted in the enhanced production and suppressive function of Treg cells, as well as upregulated Foxp3 expression, in a HDAC9-dependent manner.^385^ In contrast, HDAC5-deficient animals were not protected from transplanted tumors, likely because the CD8^+^ T cells in HDAC5^–/–^ mice released much less IFNγ. Targeting HDAC5 impairs the suppressive activity and de novo induction of Tregs, but also inhibits the ability of CD8^+^ T cells to bind to their cognate receptors.^386^ In contrast, the class-I-specific HDAC inhibitor entinostat reduces Treg cell activity, leading to enhanced antitumor immunity.^387^ These results indicate that different HDAC inhibitors have different effects on tumor immunity, suggesting the potential role of selectively targeting HDACs to improve antitumor immunity. ACY-1215, a specific inhibitor of HDAC6, inhibited the function of Treg cells and, in conjunction with JQ1, stimulated antitumor immunity.^370^

ACY-1215’s ability to target HDAC6 (class IIb) while sparing HDAC9 activity demonstrates the therapeutic potential of isoform-selective HDAC inhibitors for fine-tuning Treg gene regulation. Targeting EP300/CBP and TIP60 acetylation-dependent regulation of Foxp3 expression may be another strategy to regulate Treg function.^388,389^ Targeting the EP300/CBP bromodomain using small compounds (CP1703, CP1644, and GNE-781) reduces FOXP3 acetylation and impairs Treg differentiation, indicating that targeting the EP300/CBP bromodomain may be of potential interest for reducing Treg-mediated immunosuppression.^390,391^ EZH2 is essential for maintaining the identity of Tregs upon activation.^392^ CPI-1205, an EZH2 inhibitor, inhibits intratumor Treg function and even converts Tregs toward a TH1 cell-like phenotype with increased IFN production.^393^

Due to their inhibitory effects on CD8^+^ T-cell toxicity, pan-HDAC inhibitors have limitations. Research works should focus on the effect of subtype-selective HDAC inhibition on CD8^+^ T cells.^394^ The small-molecule HDAC3-selective inhibitor RGFP966 can significantly enhance the cytotoxic function of CD8^+^ T lymphocytes.^395^

Given the relevance of TILs in antitumor immunity, it is necessary to perform additional research into the possibility of manipulating T cells toward favorable antitumor phenotypes by targeting certain epigenetic complexes.

H3K27 trimethylation inhibition by EZH2 inhibitors (UNC1999 and EPZ005687) enhanced the expression of genes associated with NK cell cytotoxic function, such as Klrk1 (encoding NKG2D), and the in vitro cytotoxic activity of NK cells.^363^ In HCC, EZH2 inhibition with a small molecule (GSK126) may simultaneously upregulate NKG2D ligands on tumor cells, indicating that EZH2 inhibitors may promote NK cell killing by modulating both NK cells and crucial NK-activating molecules on tumor cells.^364^ GSK126 was also found to be associated with increased nfiltration MDSCs and fewer CD4^+^ and IFNγ^+^CD8^+^ T cells.^396^ Muscle-invasive bladder cancer cells with KMD6A and SWI/SNF mutations were more sensitive to an EZH2 inhibitor (EPZ011989), which is mediated by enhanced NK cell-related signaling, resulting in tumor cell differentiation and cell death.^397^

In a recent in vitro compound screening study, inhibiting the histone demethylases JMJD3/UTX with GSK-J4 decreased the expression of many proinflammatory cytokines, such as IFN, TNF, and granulocyte-macrophage colony-stimulating factor (GM-CSF), without affecting the cytotoxic killing activity of NK cells.^398^

Class-I/II/IV HDAC inhibition by panobinostat has an immune-enhancing effect in HER2^+^ breast tumors, providing compelling evidence that HDAC inhibitors enable trastuzumab to trigger an NK cell-mediated response, thus eradicating trastuzumab-refractory HER2^+^ tumors.^399^

##### RNA modification

Recently, a series of inhibitors targeting RNA modification regulators have been successfully developed.

Among them, the inhibitors developed for m^6^A modification have been studied the most. Several FTO inhibitors, both non-specific and specific, have been identified., such as rhein, MO-I-500, fluorescein, meclofenamic acid (MA), 2-hydroxylglu-tarate (R-2HG), FB23, FB23-2, and FTO-04, showing antitumor biological functions.^115,240,400–405^ However, these small molecules have shown limited clinical potential, and thus, more effective FTO inhibitors have been developed through a series of screening and validation trials, including CS1/CS2 and Dac51, which not only effectively inhibit tumor growth but also enhance T-cell toxicity.^240,241^ Blocking FTO could improve the efficacy of tumor immunotherapy. Simona Selberg and colleagues identified the inhibitory effects of two small-molecule compounds (2-{[1-hydroxy-2-oxo-2-phenylethyl]sulfanyl} acetic acid and 4-{[furan-2-yl] methyl}amino-1,2-diazinane-3,6-dione) on the proliferation of leukemia cells.^406^ Additionally, targeting ALKBH5 with a specific inhibitor (ALK-04) suppressed MDSC and Treg infiltration and enhanced the anti-PD1 therapy efficacy.^242^ STM2457, a highly potent and selective first-in-class METTL3 catalytic inhibitor, was confirmed to reduce AML growth.^407^ Thiram, an m^1^A inhibitor, was found to block the interaction of TRMT6 and TRMT61A, thus significantly reducing the m^1^A level. Importantly, it was verified that the combination of thiram with the PPARδ antagonist GSK3787 synergistically inhibits the growth of liver tumors.^408^ The effect of targeting pseudouridine in tumor therapy has recently been elucidated. Cui et al. found that a chemical inhibitor of PUS7 (C17) could block PUS7-mediated pseudouridine modification, inhibit the tumorigenesis, and extend the life span of tumor-bearing mice. This confirmed the efficacy of this inhibitor and provided preclinical evidence for potential treatment strategies for glioblastoma.^409^ A-to-I RNA editing dysregulation has been linked to a variety of cancers. Researchers have recently designed and synthesized PNAs, 2′-O-Me alone, and 2′-O-Me/PS-modified antisense oligonucleotides (ASOs) that target the editing region or editing site complementary sequence (ECS) of AZIN1. It was found that 2′-O-Me/PS-modified ASO3.2 (targeting the ECS) reduced the viability of cancer cell lines, indicating that developing an ASO-based RNA editing inhibitor holds considerable promise for cancer treatment.^410^

Collectively, the above findings demonstrate a strong potential for targeting RNA modification regulators to improve the efficacy of antitumor therapy and immunotherapy. The field of epigenetic therapies still need further study and development since the epi-drugs currently entering clinical trials mainly focus on DNA methylation and histone modification.

#### CRISPR/dcas9

As mentioned above, drugs targeting epigenetic factors have the potential to enhance antitumor immunity; however, the major drawback of these drugs is non-specificity, which in turn exhibits off-target and other side effects.^411^ CRISPR/dCas9, a recently developed CRISPR-mediated epigenome editing tool, provides an attractive alternative.^412^ CRISPR/dCas9 is an endonuclease-deficient technology that achieves good target specificity via single guide RNA (sgRNA)-based selective DNA-binding characteristics.^413^

The CRISPR/dCas9 technology has also been used extensively in the field of epigenetics. Recently, dCas9 was coupled with HAT and histone demethylase domains from p300 and LSD1, respectively, to selectively modulate local histone modifications, resulting in robust transcriptional activation or repression of target genes.^414,415^ Liu et al. created fusions of CRISPR-Cas9 with m^6^A writers and erasers to enable site-specific RNA methylation and demethylation.^416^ These engineered m^6^A writers and erasers can insert or remove m6A at particular sites without altering the basic sequence. Consequently, a more accurate dCas13 was developed.^417^

This powerful technique allows targeted and precise modulation of epigenetic factors, providing a platform for precision therapy, but more research needs to be carried out to advance epigenetic immunotherapy.

#### Adoptive cell therapy

Epigenetic therapies in combination with adoptive cell therapy (ACT) currently holds great potential, including chimeric antigen receptor T-cell (CAR T-cell) therapy.^418^

Due to advances in ACT manufacturing and administration, epigenetic treatments can be used either to prepare T cells before reinfusion or to directly target tumors. Epigenetic therapies, such as inhibitors of EZH2, LSD1, or HDAC, and hypomethylating agents, can be used to pretreat patients, creatingan inflammatory environment prior to the transfer of T cells by stimulating viral mimicry and increasing expression of antigen presentation by MHC class I on tumor cells.

Enhanced antitumor activity was observed with adoptive T-cell transfer and adjunctive use of the HDAC inhibitor LAQ824 in melanoma.^419^ JQ1, an inhibitor of BET proteins, enhances the persistence and antitumor effects of CAR T cells in murine T-cell receptor therapy models. T cells treated with JQ1 demonstrated improved in vivo persistence and antitumor activity. These findings are pertinent for the development of optimum T-cell grafts in cellular immunotherapy.^420^

As for histone methylation, treatment with an EZH2 inhibitor sensitizes Effective cytolysis of Ewing sarcoma cells by G_D2_-specific CAR-modified T cells.^421^ This strategy is expected to be administered clinically to enhance the efficacy of adoptively transferred GD2-redirected T cells against Ewing sarcoma, although in vivo validation is required. Joseph A Fraietta reported the use of targeted epigenetic therapy in combination with CAR T therapy which achieved substantial clinical significance. The researchers discovered that disruption of TET2 increased the therapeutic efficacy of T lymphocytes that targeting CD19. In conclusion, the administration of T cells derived from significant clonal expansion of a single CAR-transduced T-cell with biallelic TET2 malfunction converted a non-curative response in a 78-year-old CLL patient into a deep molecular remission.^422^ Changes in the epigenetic environment that enhance the efficacy of CAR T-cell therapy are encouraging and have important clinical implications for the delivery of bespoke cell therapies.

In addition to CAR T-cell therapy, CAR NK cell therapy is also effective for antitumor therapy.^423^ However, obtaining large numbers of NK cells in vitro in a short period of time is challenging.^424^ Current research indicates that adding cytokines (IL-2 + IL-15) or co-cultivating with K562 feeder cells significantly increases the development of NK cells in vitro, which provides a strategy to improve the efficacy of ACT therapy by combination with epigenetic modification.^423,425,426^

Overall, these results demonstrate the potential for epigenetic therapies to enhance the production of ACT cells, and we anticipate that such strategies will be systematically modified over time to confer increased benefits.

## Discussion

Despite substantial research into the biological roles of epigenetic modification in tumors in recent years, techniques for generating medicines targeting epigenetic modifications for cancer immunotherapy are still in their infancy. In this review, we highlighted current work in understanding the functions and mechanisms of epigenetic modification modulators in immune cells, as well as their effects on immunological responses in the TME.

It is also evident from recent studies that hematopoietic malignancies exhibit high sensitivity to epigenetic and immune-related therapies compared to solid tumors. Compared with hematopoietic malignancies, solid tumors are at a disadvantage because of their genomic complexity, drug exposure environment, and intratumoral heterogeneity. Therefore, in cases in which single-agent immunotherapy is not efficacious, combination epigenetic therapy may induce unexpected synergistic effects.

A recent study reported that ALKBH5 regulates lactic acid and thus leads to immune resistance during ICB treatment, which suggests that epigenetic inheritance can enhance the effects of immunotherapy.^242^ Targeting epigenetic modification induces reprogramming of tumor metabolic processes, tumor death process, and the TME remodeling, and these effects have application prospects but also are associated with challenges.

Although many drugs targeting epigenetic factors have been developed to date, epigenetic inhibitors may have adverse effects on the TME. The TME is not affected by a single epigenetic inhibitor. HDAC inhibitors, as previously mentioned, may have different effects on different immune cells. The ability of ACY-1215 to target HDAC6 (a class IIb HDAC) while retaining HDAC9 activity promotes positive regulation of antitumor immunity.^370^ As a result, more in-depth epigenetic studies are still required to clarify the characteristics of TME remodeling caused by various epigenetic modifications in order to develop highly selective epigenetic inhibitors for more efficient targeting efficiency.

Importantly, epigenetic regulators work in complex ways, and in cancer, they may be needed to maintain the expression of some key target genes. If these regulators could be universally targeted, then the balance could be thrown off, enough to cause cellular catastrophe. The CRISPR/dCas9 technology has clear advantages. The precise targeting of epigenetic modification sites greatly reduces off-target effects and other side effects. The key to a successful clinical translation is minimizing off-target effects and overcoming delivery challenges.

Advances in in-depth and precise sequencing technologies have provided better platforms for relevant research. To date, analysis of the single-cell transcriptome of immune cells has greatly enhanced our understanding of the TME. Therefore, we believe that deeper spatiotemporal analysis of the epigenetic genome at the single-cell level could complement our understanding of epigenetic modification and lead to the development of better therapeutic strategies.

The evidence linking epigenetic modification to cancer immunity strongly suggests that the development of therapies targeting epigenetic modification pathways can improve immunotherapy efficacy. However, specifically targeting epigenetics without inducing severe toxic effects remains a great challenge. Therefore, understanding the mechanisms of epigenetic modifications and learning to control them are worthy lines of further investigation. The diversity of epigenetic modifications gives hope that this is only the beginning of the era of antitumor therapy targeting RNA epigenetic factors.
